# Combination of Oral Nutritional Supplements and Dietary Counseling Is More Effective than Dietary Counseling Alone in Improving Growth Indicators among Undernourished United States Children: A Randomized Controlled Clinical Trial

**DOI:** 10.1016/j.cdnut.2026.109552

**Published:** 2026-10-09

**Authors:** Grace M Niemiro, Tricia L Hart, Jennifer A Williams, Menaka Yalawar, Geraldine E Baggs, Penni D Hicks

**Affiliations:** 1 Abbott Laboratories, Nutrition Division, Nutrition Science, Columbus, OH, United States; 2 MARS Group Inc., Bloomington, IL, United States

**Keywords:** malnutrition, pediatric, weight gain, body composition, eating behavior

## Abstract

**Background:**

Approximately 14% of United States children show ≥1 indicator of undernutrition, highlighting an opportunity to deepen research evaluating oral nutrition supplements (ONSs) on growth in United States children.

**Objectives:**

The purpose of this 2-arm, individually randomized, parallel controlled trial was to evaluate the impact of ONS plus dietary counseling (ONS+DC) compared with DC alone on growth in undernourished United States children (36–107 mo) over 120 d.

**Methods:**

Over the 120-d intervention, participants [mid-upper arm circumference (MUAC) or body mass index (BMI)-for-age *z*-score between −3 and −1) attended 2 DC sessions, and ONS+DC group ingested 2 servings of ONS (480 kcal) daily. Growth measurements occurred at baseline, days 30, 60, and 120. Parents/caregivers completed a 24-h dietary recall and questionnaires at all timepoints. A total of 279 children participated (ONS+DC: *n* = 174 enrolled, *n* = 89 protocol evaluable; DC: *n* = 105 enrolled, *n* = 82 protocol evaluable). The primary outcome was change in weight-for-age *z*-score at 120 d.

**Results:**

ONS+DC had a significantly higher change in weight-for-age *z*-score than DC at 120 d (group difference: 0.255 ± 0.058, *P <* 0.001). Average and change from baseline in weight, weight-for-age, weight-for-height, and BMI-for-age *z*-scores and percentiles were larger in ONS+DC than DC at all timepoints (*P* < 0.05). Change in MUAC-for-age *z*-score was higher in ONS+DC than DC at 30 and 120 d (*P* < 0.05). Change in height-for-age percentile was higher in ONS+DC than DC at 120 d (*P* < 0.05). Change in muscle area was larger in ONS+DC than DC over 120 d (*P* < 0.05), with no differences in arm fat area or index (*P* > 0.05).

**Conclusions:**

In undernourished United States children, 2 servings of ONS over 120 d improved growth measures more than DC alone.

This trial was registered at clinicaltrials.gov as NCT05161000 (https://clinicaltrials.gov/study/NCT05161000?intr=NCT05161000&rank=1; registered 3 December 2021).

## Introduction

Pediatric malnutrition is defined as an imbalance between nutrient requirements and intake that results in cumulative deficits of energy, protein, or micronutrients that may negatively affect growth, development, and other relevant outcomes [1]. Pediatric undernutrition is a subset of malnutrition and can manifest in wasting, stunting, underweight, and micronutrient deficiencies and can be classified as mild, moderate, or severe [2], [3]. In addition to anthropometric growth deficits, children with undernutrition may have low levels of physical activity [4], impaired mental development and school achievement, along with behavior abnormalities [5]. Long-term childhood malnutrition has been positively associated with cardiometabolic disease and risk factors, impaired cognition, and chronic disease [6], [7], [8], [9]. Beyond possible long-term health impacts, research has shown that early life undernutrition is associated with lower socioeconomic outcomes and earning potential in adulthood [10], [11]. According to the WHO, ∼149 million children under 5 were estimated to be stunted and 45 million were estimated to be wasted in 2022 across the globe [12]. Approximately two-thirds of these children have wasting with moderate undernutrition, which can result in a greater absolute number of deaths than severe undernutrition [13]. On the basis of 2015 diagnostic indicators of pediatric undernutrition, it was estimated that 14% of United States children exhibited ≥1 anthropometric indicator of childhood undernutrition, independent of race/ethnicity and income categories [14], [15], with the majority of these cases considered mild [16]. These results showcased that a nonhospitalized, general United States population (NHANES) can exhibit indicators of pediatric undernutrition at a single timepoint. Identification and preventative treatment of undernutrition at mild and moderate classifications are important to prevent deterioration into severe undernutrition, which presents higher morbidity and mortality risk [13]. Given the risks associated with long-term undernutrition, effective strategies to reduce prevalence are needed.

Previous treatment guidelines were based on the categorization of undernutrition into organic (i.e., caused by an underlying medical condition) or nonorganic/psychosocial (i.e., no medical condition is found) [19]. These 2 categories made treatment guidelines too inflexible for patient treatment because of the complex nature of pediatric undernutrition. This led to the development of the 2026 Clinical Practice Guideline (CPG) for diagnosis and management of faltering weight by the American Academy of Pediatrics (AAP) and the North American Society for Pediatric Gastroenterology, Hepatology and Nutrition, which aims to standardize practice to identify and treat early faltering weight, regardless of underlying cause, for pediatricians and other pediatric healthcare providers to minimize or avoid the development of chronic or significant undernutrition [14]. Identification of a child who is undernourished, particularly at mild (e.g., faltering weight) or moderate status, may help mitigate potential negative side effects of undernutrition later in life.

The new CPG recommends ceasing the use of sociodemographic characteristics as a risk factor, increasing caloric intake, incorporating oral nutritional supplements (ONS), and therapy in children who exhibit early signs of pediatric faltering growth [14]. These interventions are intended to help children consume a diet adequate in energy, macronutrients, and micronutrients to reduce the risk or further prognosis of poor growth and to encourage food sources with micronutrients they may be lacking [13], [20], [21]. Regardless of the cause of faltering weight, increasing caloric and micronutrient intake can provide large benefits, such as long-term positive eating habits that support optimal growth. ONS represent a dietary tool for children with faltering weight and/or undernutrition by providing supplemental macronutrients and micronutrients at sufficient levels to individuals who do not have adequate intake through table food alone. Previous trials across the globe have shown that the use of a supplemental ONS improved growth outcomes compared with other interventions, including usual diet, placebo, or dietary counseling (DC) alone [22]. To our knowledge, there have only been 2 trials conducted in the United States evaluating supplemental ONS, but they were not conducted in children with undernutrition [23], [24]. Therefore, additional clinical studies to evaluate the impact of supplemental ONS in United States children with mild to moderate undernutrition will contribute to the existing body of research.

The aim of this trial was to evaluate the effect of consuming 2 servings of supplemental ONS (480 kcal/d) and DC compared with DC alone on growth in children with ≥1 indicator for mild to moderate undernutrition in the United States for 120 d [3]. We hypothesized that consuming 2 servings of supplemental ONS (480 kcal/d) in addition to DC would improve growth outcomes, including the primary outcome of change in weight-for-age *z*-score (WAZ) at 120 d, and secondary variables of changes and absolute values of percentiles and *z*-scores in weight, height, BMI, weight-for-height, and mid-upper-arm circumference (MUAC) more than DC alone throughout 120 d (Supplemental Table 1).

## Methods

### Trial design

This was a prospective, randomized, controlled, open-label, parallel-group multicenter study conducted in the United States across 17 locations in 10 states (Supplemental Table 2) from April 2022 through June 2024. The trial lasted 4 mo (120 ± 7 d) and consisted of 4 visits on days 1, 30, 60, and 120 (Figure 1). The primary outcome of this trial was to compare the change in WAZs from baseline to 120 d between ONS+DC and DC groups at 120 d. The secondary outcomes of this trial were the following: *1*) to compare other growth indicators from baseline to all timepoints between ONS+DC and DC groups; and *2*) to compare dietary intake and appetite scores at all timepoints between the ONS+DC and DC groups. Supportive outcomes of interest between the ONS+DC and DC groups at all timepoints were the following: *a*) to compare hand grip; *b*) body composition outcomes; *c*) nutrition blood biomarkers; *d*) clinical outcomes; *e*) activity and sleep measurements; *f*) physical activity; *g*) caregiver stress; *h*) child eating behaviors; and *i*) caregiver satisfaction on child’s growth and health status. A more detailed outline of specific endpoints is listed in Supplemental Table 1.

**FIGURE 1:**
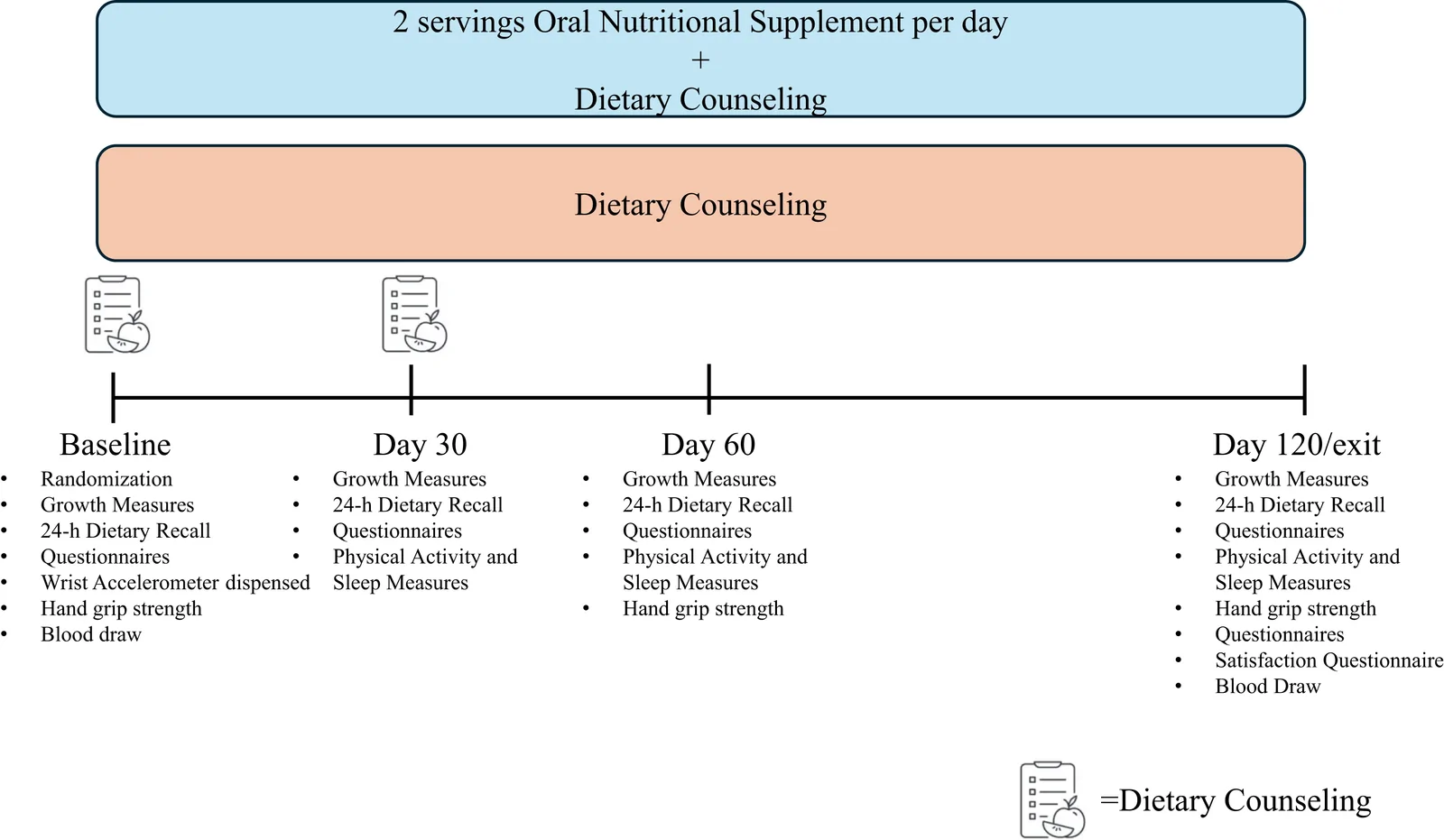
Schematic of study timeline. ONS, oral nutrition supplement.

Because of the nature of the intervention, only outcome assessors were blinded to randomization. Initially, eligible participants were randomly assigned in a 1:1 ratio until 80% of participants were enrolled, followed by a preplanned adjustment to 10:1 (ONS+DC: DC) to account for higher attrition in the intervention group related to increased participant burden and noncompliance. Randomization of participants was assigned using a prospective, computer-generated randomization plan with a biased-coin minimization approach to randomization that dynamically assigned children to maintain the best possible balance within strata, within site, and across sites overall. An electronic data capture system was utilized to assign subject numbers and randomly divide participants to treatment groups according to the generated randomization schedules. Randomization was stratified by gender, age group (36–71 mo, 15 d and 71 mo, 16 d–107 mo), and undernourished classification category used for eligibility criterion [BMI-for-age *z*-score (BMIAZ), MUAC-for-age *z*-score; Centers for Disease Control (CDC) guidelines) at each site and across sites to minimize confounding effects. Children who qualified were assigned to either the ONS+DC group or DC only group.

Both groups attended 2 DC sessions conducted by registered dietitians. The ONS+DC group was also provided with a pediatric supplemental ONS that was clinically labeled to mask identity (retail PediaSure Grow & Gain; 8 fl oz; Abbott Nutrition) and was instructed to consume 2 per day orally. The DC group did not receive study product and hence, the study was unblinded. The study site personnel were not blinded to the group the participant was in to encourage participation relevant to each participant and their family, when needed. All individuals involved in data analysis were blinded. Both groups were instructed to abstain from nonstudy ONS during the study period. All primary, secondary, and supportive outcome measures were assessed at baseline and day 120 or study exit. This study was approved by a central Institutional Review Board (WCG IRB). One site also submitted to their local IRB (Wake Forest School of Medicine IRB). The child’s legal guardian provided written informed consent and completed a Health Insurance Portability and Accountability Act form before enrollment. This trial was preregistered at clinicaltrials.gov (ID: NCT05161000). The protocol was conducted in accordance with the Declaration of Helsinki and good CPGs along with applicable laws and regulations.

### Participants

Children between 36 and 107 mo of age who were undernourished, defined by BMIAZ between −3 and −1 or MUAC-for-age *z*-score between −3 and −1 [3], [25], were eligible for this trial. MUAC is an index of nutritional status for children and 1 of the 4 indicators of pediatric undernutrition [3], [26], [27], [28]. Participants were excluded if the child had a height-for-age *z*-score (HAZ) ≥0, had allergies to ONS ingredients or disliked the study product, experienced chronic infections or gastrointestinal disorders, or had any clinically significant medical condition per clinician’s judgment. To limit confounding variables, participants who currently consumed ONS (i.e., ≥15 d in the past month) or were enrolled in a concomitant trial were excluded. A taste test of the products was conducted before enrollment to ensure acceptance of ONS. Participants were recruited through a central recruitment campaign (Study Kick), referral from their own primary care physician who was familiar with the study, and/or their primary care physician was an active investigator at a study site. If participants and their families were interested, research sites would help determine the participant’s eligibility through screening questions before consent and enrollment.

### Intervention

Caregivers and participants in both groups received DC from a registered dietitian over 2 sessions at visits 1 and 2 (days 1 and 30). Session content was guided by USDA dietary recommendations [29] in combination with standard practice of clinic sites (e.g., length of visit) by the dietitian. Specifically, counseling centered on messages of a balanced diet and the amounts of various nutrients needed in the daily diet.

In the ONS+DC group, participants could select 2 from 4 different flavors of supplemental ONS (Vanilla, Chocolate, Banana, and Strawberry). Caregivers were provided with a monthly supply of ONS at each visit and instructed to provide their child with 2 ONS per day with no specific recommendations on timing. One serving of ONS (8 fl oz, 1.0 kcal/mL) provided 240 kcal, 7 g protein, 33 g carbohydrate, and 9 g fat and had ≥10% of the United States recommended daily value for 27 vitamins and minerals [30], with the full nutrient panel and ingredient list supplied in Supplemental Table 3. Caregivers were instructed to track their child’s consumption of the ONS on product diaries and return unopened supplements at study visits to evaluate compliance. Flexibility in study visits ±7 d was permissible to accommodate schedules. If a study visit was changed, the subsequent study visits remained as scheduled to meet the study duration of 120 ± 7 d.

Compliance of ONS consumption was determined by dividing the number of bottles consumed by the number of bottles participants were instructed to consume and multiplying by 100 to calculate percent adherence based on daily records. Adherence to the study protocol was classified as consuming ≥75% of the product over the 120-d period. Participants in both groups were required to attend 2 DC sessions to be considered compliant.

### Outcome assessments

#### Anthropometrics

All measuring devices were provided by the study sponsor to each site. Anthropometric measurements including body weight (kg), height (cm), triceps skinfold (TSF, mm), and MUAC (cm) were measured at all 4 study visits by trained study personnel. Each measurement was performed a minimum of 2 times and a third time if the first 2 measures were outside predefined limits (i.e., >100 g for weight, >0.7 cm for height, >0.2 cm for MUAC, and >1 mm TSF).

Body weight was measured in light clothing and without shoes using a calibrated weight scale to the nearest 0.1 kg. Height was measured without shoes using a stadiometer. Sites used their own equipment if it fulfilled the protocol criteria. If necessary, a Seca 807 scale and/or Seca stadiometer was provided (Seca GmbH & Co. KG). Children were given instructions to stand up straight, look straight ahead with heels together against the back of the stadiometer. Caregivers assisted with positioning. Research personnel rested the movable headboard on the participant’s head, and the measurement was recorded to the nearest 0.1 cm. Weight, height, BMI, and weight-for-height percentiles and *z*-scores were calculated based on the child’s age and sex according to CDC 2000 standards at the time measurements were taken.

Research personnel used an Abbott MUAC *z*-score tape according to manufacturer instructions. Briefly, the green arrows mark 0 on the tape, and the tail end of the tape inserts into a slit to create a loop. The midpoint between the acromion and olecranon processes was identified on the child’s left arm, the looped device was placed, and MUAC was recorded and used to calculate *z*-score. The same midpoint was used for TSF thickness. Trained research personnel firmly grasped the skin between the thumb and index finger using a skinfold caliper (Harpenden; Baty International). The skinfold was lifted 1 cm and recorded (to the nearest millimeter) with the caliper 4 s after the caliper pressure was released. To estimate total body fat and muscle mass, TSF and MUAC measures were used to derive arm muscle area (AMA), arm fat area (AFA), and arm fat index (AFI). The calculations [31], [32] were as follows: AMA = [MUAC − (TSF × π)]^2^/(4 × π); AFA = total arm area (TUA) − AMA, where TUA = MUAC^2^/(4 × π) and AFI = (AFA/TUA) × 100.

#### Handgrip strength

Handgrip strength was measured using the child’s dominant hand with a handgrip strength dynamometer (Jamar Plus+; Performance Health). The child’s arm was placed by their side, and the elbow was flexed 90° with their forearm in a neutral position. The child performed 2 maximal force grasps each lasting 3–4 s to provide an average. This was repeated 3 times, and the mean grip force of the 3 trials was used as the final strength value. The force was measured in kilograms. Children received verbal encouragement throughout each trial.

#### Micronutrient biomarkers

After a ≥8-h overnight fast, blood samples were collected in both groups on day 1 and day 120 in serum separator tubes by trained nurses. If participants were unfasted, it was noted, and if possible, blood draws were rescheduled. Samples were centrifuged down for their respective protocol, aliquoted, and frozen at −20°C or colder until shipment. Frozen samples were shipped on dry ice to Q^2^ Solutions (Valencia, CA; IQVIA Laboratories) for analysis of 25-hydroxyvitamin D, vitamin K, vitamin A (retinol), vitamin B12, vitamin E (α-tocopherol), and folate. If samples were not obtained at visit 1, blood was not collected at visit 4.

#### Dietary intake

Dietary intake was assessed by a single 24-h dietary recall reported by a caregiver at all 4 visits. The 24-h dietary recalls were conducted at each study visit at the site. Dietary diversity score was calculated by summing the number of food groups defined by What We Eat in America with 1 point per food group consumed over the 24-h period [33]. A maximum score of 8 was possible.

#### Activity and sleep

At baseline, a commercially available movement tracker (Garmin VivoFit Jr3; Garmin International, Inc.) was fitted on the child’s wrist to track sleep and physical activity data throughout the trial. The device was linked to the caregiver’s phone for the duration of the study. Average time per day or night in minutes were collected at each visit and an average of the past 7 d (with a minimum of 5 d if 7 were not available) were used to estimate activity level and sleep time, respectively. If participants were unable to keep the device on at all times, study personnel encouraged parents/caregivers to have children wear the device for ≥5 d before the next visit. Children were allowed to keep the device after the end of the trial.

#### Questionnaires

Three questionnaires were administered at all timepoints to caregivers: *1*) visual analog scale (VAS) of perception of child’s appetite over the last 24 h (0–10, 0 = ate very little, 10 = ate a lot); *2*) VAS of perception of child’s activity over the last 24 h (1–10, with 10 reflecting more activity); and *3*) caregiver reported sleeping habits of the child. Caregivers reported illnesses, daycare, and healthcare provider visits of their child for clinical outcomes of interest at all timepoints. At baseline, day 60, and day 120/exit, caregivers completed 2 validated questionnaires: *1*) the Parental Stress Scale [34] and *2*) Childhood Eating Behavior Questionnaire (CEBQ) [35]. At 120 d, caregivers reported opinions about their child’s growth with 2 Likert scale questions and one VAS question.

### Adverse events and clinical outcomes

Adverse events (AEs) were defined as any untoward medical occurrence in a clinical study participant that does not necessarily have a causal relationship with a study product. An AE can therefore be any unfavorable and unintended sign (including clinically significant abnormal finding), symptom, or disease temporally associated with the study, whether or not related to the study product. An AE can be either nonserious (NSAE) or serious (SAE). An SAE was defined as any untoward medical occurrence that results in death, is life-threatening, requires inpatient hospitalization or prolongation of existing hospitalization, results in persistent or significant disability or incapacity, or a congenital anomaly or birth defect. Other important medical events requiring medical or surgical intervention to prevent a serious outcome were also considered SAEs. All other AEs were defined as NSAEs.

AE coding and grouping in this study utilized the Medical Dictionary for Regulatory Activities (versions ranging from 25.0 to 27.0) terminology. AEs were collected by a standard method and were reported by parents and caregivers and recorded by the study sites. Both NSAEs and SAEs were medically confirmed and assessed for intervention causality by the study site physicians. Intervention causality utilized the following definitions and was assessed for all AEs (NSAE or SAE): *a*) probable relationship to study product (the AE has a strong temporal relationship to the study product or recurs on rechallenge and another etiology is unlikely or significantly less likely); *b*) possible relationship to the study product (the AE has a strong temporal relationship to the study product and an alternative etiology is equally or less likely compared with the potential relationship to the study product); *c*) probably not related to study product (the AE has little or no temporal relationship to the study product and/or a more likely alternative etiology exists); and *d*) not related to study product (the AE is due to an underlying or concurrent illness or injury and is not related to study product). AEs were collected from the time the informed consent form was signed and until 2 d after study exit/early exit. Participants’ parent/caregiver received an exit contact 3–7 d after exit/early exit to assess if there were any new changes to AEs ongoing at exit/early exit; and/or if any new AEs occurred within the 2 d after exit/early exit. Participants with events ongoing at exit/early exit required follow-up per the following: *1*) AEs were followed for ≤7 d postexit; and *2*) SAEs were followed until the event was resolved or a reasonable explanation was identified.

### Statistical analysis

The sample size was determined to detect a clinically significant difference of 0.24 between groups for the change in WAZ, the primary outcome. Assuming an SD of 0.52 based on prior data, a total of 75 participants per group was required to provide 80% power using a 2-sided α = 0.05 level *t*-test. With attrition estimates of 52% for ONS+DC and 28% for DC, the target enrollment was 275 participants.

All statistical analyses were conducted in SAS (version 9.4; SAS Institute Inc.) and alpha was set at 0.05, except for interaction effects, which were set as 0.10 level tests. In the presence of significant interaction effects of study group with stratification variables, Holm’s step-down Bonferroni procedure was used to adjust for multiple comparisons. *P* value adjustments for multiplicity of testing secondary outcomes measured at several timepoints was also made using Holm’s step-down Bonferroni procedure. In the absence of statistical significance, results were also interpreted for how meaningful the difference was as measured by Cohen’s *d*: small effect = 0.2, medium effect = 0.5, large effect = 0.8. In addition, categorical outcomes where the gap between groups was ≥10% were noted. All randomly divided participants who received ≥1 study feeding in the ONS+DC group and all randomly divided participants in the DC group were included in the intent-to-treat (ITT) dataset and were included in all analyses. There was no imputation of missing data. Instead, parametric models fit to all available data based on likelihood methods that account for gaps in the observations were employed. A summary of possible model factors used in analyses included study center, study group, gender, age group, age group∗study group, gender∗study group, visit, and study group∗visit. Specific model factors are fully specified for each analysis in the table and figure legends. For all repeated measures analysis of covariance (ANCOVA), and ANCOVA at each timepoint, the corresponding baseline value was used as a covariate, if collected. Height-related analyses (repeated measures ANCOVA and ANCOVA) on HAZ, height-for-age percentile (HAP), and height additionally included age at study day 1 and number of children <18 y old in the household (for change in HAZ only) as covariates. An additional analysis of protocol-evaluable participants was performed to gauge efficacy of ONS+DC compared with DC in children who were adherent and completed the trial. Height values that were biologically implausible for extreme growth were excluded from the analysis. For micronutrient analyses, if a participant fell outside of the sensitivity ranges, the following values were used: folate values >48 ng/mL were defined as 48 ng/mL, vitamin K values <50 pg/mL were defined as 25 pg/mL, and vitamin K values >2500 pg/mL were defined as 2500 pg/mL.

The primary outcome, change in WAZ from baseline to 120 d, was evaluated via ANCOVA. ANCOVA was also used for secondary and supportive outcomes including the change from baseline to each timepoint for weight-for-age percentile (WAP), HAZ and HAP, weight (kg), height (cm), weight-for-height *z*-score (WHZ) and percentile (WHP), BMIAZ and BMI-for-age percentile (BMIAP), MUAC-for age *z*-score (MUACAZ), and serum micronutrients. Secondary and supportive outcomes including all mentioned above and AFI, AMA, AFA, handgrip strength (kg), dietary diversity, appetite score, sleep data (questionnaires and wrist measurements), activity data (wrist measurements), physical activity score, caregiver stress level, and child eating behavior questionnaire subscales were analyzed via repeated measures ANCOVA. Caregiver opinion data were analyzed using ANOVA or Fisher’s exact test. The incidence of acute illnesses was analyzed by the chi-square test. The number of AE reports of acute illnesses as well as duration/days/number of clinical outcomes of interest was analyzed using Wilcoxon rank sum test at each timepoint. Incidence (Y/N) of clinical outcomes of interest was analyzed by Fisher’s exact test at each timepoint. Model assumptions and goodness of fit were verified during analysis. Data shown in tables reflecting inferential results were given as least squares means (LSM) ± SEM, or median (1st quartile, 3rd quartile) for continuous outcomes and *n* (%) for categorical outcomes.

## Results

### Participants

In total, 279 participants were enrolled and randomly assigned (Figure 2) across the 17 study sites. Because 1 participant did not receive any product, 278 participants were included in the ITT cohort and 171 were protocol evaluable (PE). Results of ITT and PE analyses were similar and only ITT results are presented here, unless otherwise noted. Participant characteristics and growth-associated variables at study entry were similar between groups (Tables 1 and 2 and Supplemental Table 4). Adherence to ONS intake in the ONS+DC group was high (median of 87%), and both groups had 90% DC compliance.

**FIGURE 2:**
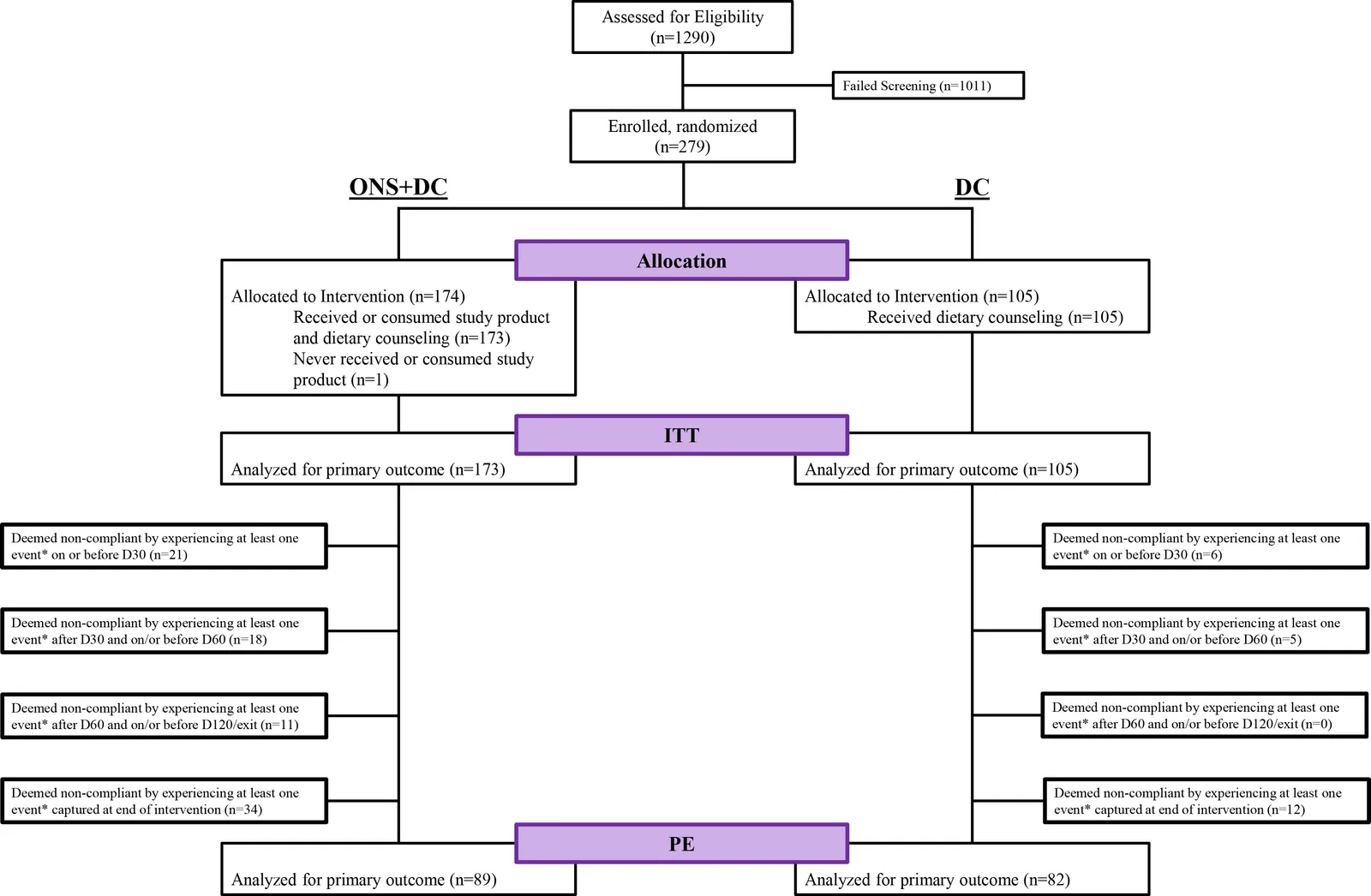
CONSORT diagram. DC, dietary counseling; ITT, intent-to-treat; ONS+DC, oral nutrition supplement + dietary counseling; PE, protocol evaluable. ∗A participant was classified as protocol noncompliant for the analysis if 1 or more of the events described below occurred: *1*) Participant entered the study and did not satisfy eligibility criteria; *2*) Participant received wrong intervention at the time of randomization, contrary to the randomization scheme; *3*) Participant had missing primary outcome data; *4*) Participant did not receive 2 sessions of dietary counseling; *5*) Participant consumed excluded concomitant treatment defined as: *a*) Participants consumed nonstudy ONS, protein shakes, or nutrition supplements that could profoundly affect study outcomes for ≥30 d over the 120-d intervention period, which is equivalent to 25% of the intervention period; *b*) Participants consumed medications or supplements that could profoundly affect study outcomes for ≥30 d over the 120-d intervention period, which is equivalent to 25% of the intervention period. If the child is on a multivitamin or similar supplement at enrollment, he or she should continue to consume during the study. *6*) (If randomly assigned to treatment group only) Participant did not consume the study product for >7 consecutive days during the intervention period; *7*) (If randomly assigned to treatment group only) Participant consumed <75% of prescribed study product over the 120-d intervention period.

**TABLE 1:** Participant characteristics at study entry by group^1^

	ONS+DC	DC
Sex^2^, *n* (%)		
Male	94 (54.3)	61 (58.1)
Female	79 (45.7)	44 (41.9)
Hispanic/Latino or Not Hispanic/Latino^3^, *n* (%)		
Hispanic or Latino	92 (53.2)	62 (59.0)
Not Hispanic or Latino	78 (45.1)	43 (41.0)
Not reported	3 (1.7)	0 (0.0)
Race^3^, *n* (%)		
Asian	6 (3.5)	4 (3.8)
Black or African American	15 (8.7)	11 (10.5)
White	134 (77.5)	85 (81.0)
Other	18 (10.4)	5 (4.8)
Annual household income level^3^, *n* (%)		
<$25k	17 (9.8)	13 (12.4)
>$25 but <$50k	25 (14.5)	16 (15.2)
>$50 but <$100k	56 (32.4)	26 (24.7)
>$100k	24 (13.9)	17 (16.2)
No response	51 (29.5)	33 (31.4)
Mother’s height^4^ (in)		
LSM ± SEM	63.7 ± 0.3	63.8 ± 0.4
*n*	172	104
Father’s height^4^ (in)		
LSM ± SEM	68.5 ± 0.4	68.3 ± 0.5
*n*	165	102
Age on study day 1^5^ (y)		
LSM ± SEM	6.0 ± 0.2	6.1 ± 0.2
*n*	173	105
Age group (y) on study day 1, *n* (%)		
3	33 (19.1)	26 (24.8)
4	30 (17.3)	14 (13.3)
5	30 (17.3)	18 (17.1)
6	27 (15.6)	18 (17.1)
7	36 (20.8)	15 (14.3)
8	17 (9.8)	14 (13.3)
*n*	173	105

Abbreviations: CI, confidence interval; DC, dietary counseling; LSM, least squares mean; ONS+DC, oral nutrition supplement + dietary counseling.

1 Intent-to-treat data are presented as LSM ± SEM.

2 Analyzed by Chi-square test.

3 Analyzed by Fisher’s exact test.

4 Analyzed by analysis of variance (study center, study group).

5 Analyzed by ANOVA (study center, gender, study group).

**TABLE 2:** Baseline values for growth-associated outcomes by group^1^

	ONS ± DC		DC	
	*n*	LSM ± SEM	*n*	LSM ± SEM
Weight-for-age *z*-score2	173	−1.5 ± 0.1	105	−1.8 ± 0.1
Weight-for-age percentile^2^	173	12.4 ± 1.2	105	9.8 ± 1.4
Weight (kg)	173	17.2 ± 0.2	105	16.8 ± 0.3
Height-for-age *z*-score^2^	173	−0.8 ± 0.1	104	−1.0 ± 0.1
Height-for-age percentile^2^	173	28.1 ± 1.6	104	23.7 ± 2.1
Height (cm)	173	109.7 ± 0.5	104	108.5 ± 0.7
Weight-for-height *z*-score^3^	146	−1.6 ± 0.1	90	−1.8 ± 0.2
Weight-for-height percentile^2^	146	13.9 ± 1.8	90	11.8 ± 2.1
BMI-for-age *z*-score^2^	173	−1.6 ± 0.1	105	−1.8 ± 0.1
BMI-for-age percentile^2^	173	13.6 ± 1.7	105	11.5 ± 1.9
MUAC-for-age *z*-score^3^	172	−1.4 ± 0.6	105	−1.4 ± 0.8
Arm muscle area (mm∗mm)	159	1495.7 ± 22.1	101	1501.2 ± 25.8
Arm fat area (mm∗mm)	159	616.4 ± 17.4	101	598.7 ± 20.3
Arm fat index (%)	159	29.2 ± 0.7	101	28.5 ± 0.8

Abbreviations: DC, dietary counseling; LSM, least squares mean; MUAC, mid-upper arm circumference; ONS+DC, oral nutrition supplement + dietary counseling.

1 Intent-to-treat data are presented as LSM ± SEM analyzed by analysis of variance (study center, study group, gender, age group).

2 Using CDC 2000 standards.

3 Using 1999–2012 CDC standards.

### Anthropometrics

Changes in anthropometric measures at each timepoint are presented in Table 3 and Figure 3. The primary variable, change in WAZ from baseline to 120 d (Figure 3A), was significantly higher in the ONS+DC group compared with the DC group in both ITT [LSM ± SE: ONS+DC: 0.40 ± 0.05 compared with DC: 0.14 ± 0.05; *P <* 0.0001; mean difference 0.26 ± 0.06 (LSM ± SE); *P* < 0.001] and PE cohorts (ONS+DC: 0.42 ± 0.06 compared with DC: 0.17 ± 0.06, *P* = 0.0004). These effects were significantly driven by males. Similarly, the change from baseline in WAZ was significantly higher in ONS+DC compared with DC at each timepoint and across timepoints (Table 3).

**TABLE 3:** Change in growth-associated outcomes by group^1^

Outcome	Timepoint	ONS+DC		DC		Effect size	*P* value	Adjusted *P* value
		*n*	LSM ± SEM	*n*	LSM ± SEM	Group Difference (DC − ONS+DC; LSM ± SEM)		
Weight-for-age *z*-score^2^^,^^3^	Δ Day 30	148	0.26 ± 0.03	91	0.13 ± 0.03	−0.13 ± 0.04	<0.001^4^	<0.001^4^
	Δ Day 60	130	0.36 ± 0.04	85	0.16 ± 0.05	−0.21 ± 0.05	<0.0001^4^	<0.0001^4^
	Δ Day 120	135	0.40 ± 0.05	92	0.14 ± 0.05	−0.26 ± 0.06	<0.0001^4^	<0.0001^4^
Weight-for-age percentile^2^^,^^3^	Δ Day 30	148	4.87 ± 0.63	91	1.62 ± 0.75	−3.24 ± 0.82	0.0001^4^	0.00001^4^
	Δ Day 60	130	7.67 ± 0.92	85	2.61 ± 1.04	−5.06 ± 1.1	<0.0001^4^	<0.0001^4^
	Δ Day 120^5^	135	9.17 ± 1.32	92	2.38 ± 1.42	−6.8 ± 1.5	<0.0001^4^	<0.0001^4^
Weight^3^ (kg)	Δ Day 30	148	0.66 ± 0.06	91	0.37 ± 0.07	−0.28 ± 0.08	<0.001^4^	<0.001^4^
	Δ Day 60	130	1.07 ± 0.08	85	0.59 ± 0.09	−0.48 ± 0.1	<0.0001^4^	<0.0001^4^
	Δ Day 120	135	1.51 ± 0.11	92	0.81 ± 0.12	−0.7 ± 0.13	<0.0001^4^	<0.0001^4^
Height-for-age *z*-score^2^	Overall^6^	415^7^	0.09 ± 0.02	265^7^	0.04 ± 0.03	−0.05 ± 0.03	0.0965	
	Δ Day 30^3^	149	0.04 ± 0.02	90	0.01 ± 0.0.03	−0.04 ±.0.03	0.2372	0.4744
	Δ Day 60^3^	131	0.10 ± 0.03	84	0.07 ± 0.03	−0.03 ±.0.04	0.4778	0.4778
	Δ Day 120^3^	135	0.09 ± 0.04	91	0.01 ± 0.04	−0.08 ±.0.04	0.0575	0.1725
Height-for-age percentile^2^	Overall^6^	415^7^	3.02 ± 0.67	265^7^	1.17 ± 0.80	−1.85 ± 0.87	0.0334^4^	
	Δ Day 30^3^	149	2.07 ± 0.68	90	0.81 ± 0.82	−1.26 ±.0.89	0.1556	0.3112
	Δ Day 60^3^	131	2.85 ± 0.89	84	2.19 ± 1.02	−0.67 ±.1.08	0.5394	0.5394
	Δ Day 120^3^	135	2.98 ± 1.10	91	−0.19 ± 1.20	−3.18 ±.1.28	0.0140^4^	0.042^4^
Height (cm)	Overall^6^	415^7^	1.62 ± 0.11	265^7^	1.38 ± 0.13	−0.24 ± 0.14	0.0895	
	Δ Day 30^3^	149	0.70 ± 0.11	90	0.54 ± 0.13	−0.15 ±.0.14	0.2763	0.5526
	Δ Day 60^3^	131	1.53 ± 0.15	84	1.39 ± 0.17	−0.14 ±.0.18	0.4435	0.5526
	Δ Day 120^3^	135	2.47 ± 0.19	91	2.07 ± 0.21	−0.40 ±.0.22	0.0693	0.2079
Weight-for-height *z*-score^2^^,^^3^	Δ Day 30	125	0.38 ± 0.06	77	0.14 ± 0.07	−0.24 ± 0.08	0.004^4^	0.004^4^
	Δ Day 60	105	0.57 ± 0.08	72	0.19 ± 0.09	−0.38 ± 0.10	<0.001^4^	< 0.001^4^
	Δ Day 120	110	0.56 ± 0.09	78	0.15 ± 0.11	−0.41 ± 0.12	0.001^4^	0.003^4^
Weight-for-height percentile^2^^,^^3^	Δ Day 30	125	7.68 ± 1.16	77	3.12 ± 1.48	−4.56 ± 1.63	0.006^4^	0.0018^4^
	Δ Day 60	105	9.21 ± 1.59	72	3.53 ± 1.96	−5.68 ± 2.18	0.01^4^	0.01^4^
	Δ Day 120	110	12.08 ± 2.16	78	4.05 ± 2.54	−8.03 ± 2.87	0.006^4^	0.0018^4^
BMI-for-age *z*-score^2^^,^^3^	Δ Day 30	148	0.38 ± 0.05	91	0.15 ± 0.07	−0.23 ± 0.07	0.001^4^	0.003^4^
	Δ Day 60	130	0.52± 0.07	85	0.12 ± 0.08	−0.40 ± 0.09	<.0001^4^	<0.0001^4^
	Δ Day 120	135	0.56 ± 0.10	92	0.12 ± 0.11	−0.44 ± 0.12	<0.001^4^	<0.001^4^
BMI-for-age percentile^2^^,^^3^	Δ Day 30	148	7.58 ± 1.11	91	3.24 ± 1.32	−4.34 ± 1.44	0.003^4^	0.006^4^
	Δ Day 60	130	9.24 ± 1.68	85	3.16 ± 1.91	−6.08 ± 2.02	0.003^4^	0.006^4^
	Δ Day 120	135	12.66 ± 2.23	92	4.36 ± 2.40	−8.3 ± 2.56	0.001^4^	0.003^4^
MUAC-for-age *z*-score^3^^,^^8^	Δ Day 30	147	0.22 ± 0.03	91	0.14 ± 0.04	−0.09 ± 0.04	0.0396^4^	0.0792
	Δ Day 60	130	0.30 ± 0.05	85	0.19 ± 0.05	−0.10 ± 0.06	0.0673	0.0792
	Δ Day 120	135	0.26 ± 0.07	92	0.47 ± 0.06	−0.21 ± 0.07	0.0028^4^	0.0084^4^
Arm muscle area^6^ (mm∗mm)	Overall	408^7^	108.38 ± 12.68	267^7^	65.51 ± 14.87	−42.86 ± 16.41	0.0096^4^	
	Δ Day 30	145	55.46 ± 12.65	91	27.87 ± 15.08	−27.58 ± 16.68	0.0995	0.199
	Δ Day 60	129	89.37 ± 13.50	84	60.54 ± 15.98	−28.83 ± 18.0	0.1106	0.199
	Δ Day 120	134	180.30 ± 18.78	92	108.13 ± 22.32	−72.17 ± 27.12	0.0083^4^	0.025
Arm fat area^6^ (mm∗mm)	Overall	408^7^	64.48 ± 8.44	267^7^	55.36 ± 9.94	−9.12 ± 10.94	0.4053	
	Δ Day 30	145	29.83 ± 8.15	91	34.23 ± 9.64	4.4 ± 10.51	0.6761	0.9243
	Δ Day 60	129	60.58 ± 9.15	84	51.52 ± 10.88	−9.06 ± 12.30	0.4621	0.9243
	Δ Day 120	134	103.03 ± 10.56	92	80.32 ± 12.55	−22.71 ± 14.75	0.1251	0.375
Arm fat index^6^ (%)	Overall	408^7^	0.59 ± 0.34	267^7^	0.85 ± 0.39	0.27 ± 0.42	0.5288	
	Δ Day 30	145	0.22 ± 0.35	91	0.76 ± 0.42	0.55 ± 0.47	0.2404	0.7213
	Δ Day 60	129	0.73 ± 0.37	84	0.82 ± 0.43	0.09 ± 0.49	0.8509	1.000
	Δ Day 120	134	0.82 ± 0.38	92	0.97 ± 0.44	0.16 ± 0.50	0.7519	1.000

Abbreviations: Δ, change from baseline; LSM, least squares mean; ONS+DC, oral nutrition supplement + dietary counseling.

1 Intent-to-treat data are presented as LSM ± SEM.

2 Using CDC 2000 standards.

3 Analyzed by analysis of covariance (ANCOVA) (study group, study center, age group, age group∗study group, gender, gender∗study group) with respective baseline as covariate at each timepoint.

4 *P* values indicate significance (*P* < 0.05).

5 A significant treatment∗gender interaction showed that this was driven by males (ITT males: ONS+DC: 0.47 ± 0.06, *n* = 73 compared with DC: 0.09 ± 0.07, *n* = 54; *P <* 0.001; PE males: ONS+DC: 0.47 ± 0.07, *n* = 48 compared with DC: 0.09 ± 0.08, *n* = 49; *P <* 0.001).

6 Analyzed by repeated measures ANCOVA (study center, gender, study group, study group∗gender, visit, study group∗visit, age group, study group∗age group) with respective baseline measure, age at study day 1 (HAZ, HAP, and height), and number of children <18 y old in household (HAZ) as covariate.

7 The *n* is the sum of all participants at 30, 60, and 120 d.

8 Using 1999–2012 CDC Standards.

**FIGURE 3:**
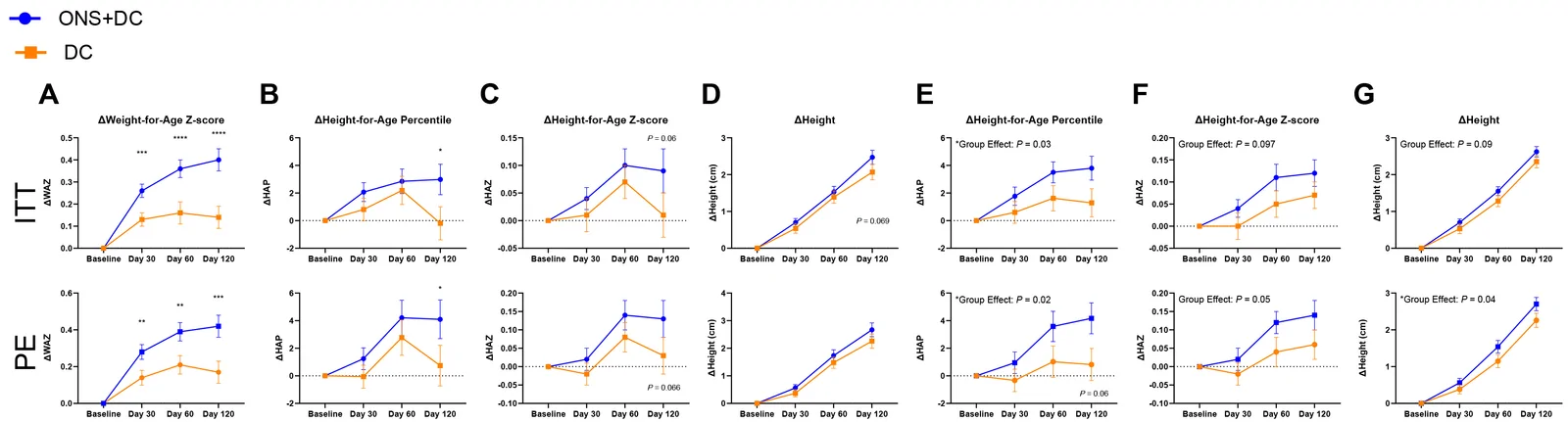
Changes in weight-for-age *z*-score and height-associated variables (intent-to-treat and protocol evaluable data are presented as LSM ± SEM). (A) Analyzed by ANCOVA (study group, study center, age group, age group∗study group, gender, gender∗study group) with respective baseline as covariate at each timepoint. (B–D) Analyzed by ANCOVA (study group, study center, age group, age group∗study group, gender, gender∗study group) with baseline value as covariate. (E–G) Analyzed by repeated measures ANCOVA (study center, gender, study group, study group∗gender, visit, study group∗visit, age group, study group∗age group) with respective baseline value, age at study day 1, and number of children <18 y old in household (HAZ) as covariate. Δ, change from baseline; ANCOVA, analysis of covariance; DC, dietary counseling; HAP, height-for-age percentile; HAZ, height-for-age *z*-score; LSM, least squares mean; ONS+DC, oral nutrition supplement + dietary counseling; WAZ, weight-for-age *z*-score. ∗Unadjusted *P* < 0.05, ∗∗unadjusted *P* < 0.01, ∗∗∗unadjusted *P* < 0.001, ∗∗∗∗unadjusted *P* < 0.0001.

Given the time frame of the intervention, height results on both the ITT and PE cohorts are presented (Table 3, Figure 3). Height analyses included HAP, HAZ, and height (cm). The change from baseline in HAP was significantly higher in ONS+DC compared with DC at 120 d in both cohorts (Table 3, Figure 3A). Repeated measures analyses showed that the change from baseline in HAP was significantly higher in ONS+DC compared with DC across timepoints in both cohorts (significant main effect of group, Figure 3D). The change from baseline in HAZ tended to be higher in ONS+DC compared with DC at 120 d but did not reach significance between groups in both cohorts (Table 3, Figure 3B). Repeated measures analyses also showed that the change from baseline in HAZ across timepoints tended to be higher in ONS+DC across all timepoints but did not reach significance in both cohorts (Table 3, Figure 3E). The change from baseline in height (cm) tended to be higher in ONS+DC compared with DC at 120 d in the ITT cohort (Table 3, Figure 3C), but not the PE cohort (Figure 3C). Repeated measures analyses showed that the change from baseline in height (cm) was significantly higher in ONS+DC compared with DC across timepoints (significant main effect of group) in the PE cohort (Figure 3F). Repeated measures analyses showed a trend for higher changes in ONS+DC compared with DC in height (cm) across timepoints in the ITT cohort but did not reach full significance (Table 3, Figure 3F).

The change from baseline in absolute weight (kg), WAP, WHP, BMIAZ, and BMIAP was significantly higher in ONS+DC compared with DC at all timepoints (Table 3). The change from baseline in WHZ was significantly higher in ONS+DC compared with DC at all timepoints (Table 3). In the PE cohort, results differed slightly with the change in WHZ not significantly higher at 60 d with ONS+DC compared with the DC group (ONS+DC: 10.90 ± 2.51, *n* = 63 compared with DC: 5.68 ± 2.40, *n* = 63; *P* = 0.0574).

The change in MUACAZ was significantly higher in DC + ONS compared with DC alone at days 30 and 120 only, and no difference on day 60 (Table 3). However, in the PE cohort, the change in MUACAZ at 60 d in the ONS+DC group was significantly higher in the DC group (ONS+DC: 0.36 ± 0.05, *n* = 79 compared with DC: 0.24 ± 0.05, *n* = 73; *P* = 0.0373).

There was a significantly higher change in AMA in ONS+DC compared with DC across all timepoints (significant main effect of group, Table 3), and also at 120 d (Table 3). Additionally, in the PE cohort, a significantly higher change in AMA in the ONS+DC compared with the DC group in AMA also occurred at 30 d (ONS+DC: 78.76 ± 15.50, *n* = 82 compared with DC: 15.83 ± 15.83, *n* = 76; *P* = 0.0011) and 60 d (ONS+DC: 110.28 ± 16.11, *n* = 79 compared with DC: 53.80 ± 16.45, *n* = 72; *P* = 0.003). There were no differences between groups at any timepoint for changes in AFA or AFI (Table 3).

Cohen’s *d* was calculated for statistically nonsignificant outcomes. A value of ≥0.2 was observed for the following variables at the following timepoints: *a*) change in HAZ at 60 (*d* = 0.24) and 120 d (*d* = 0.30); *b*) change in HAP at 30 d (*d* = 0.21); *c*) change in height at 120 days (*d* = 0.27); *d*) change in MUACAZ at 30 d (*d* = 0.30) and 60 days (*d* = 0.28); change in AMA at 60 d (*d* = 0.24); and change in AFA at 120 d (*d* =0.25).

### Micronutrients

No differences between dietary diversity and appetite occurred between groups at baseline, or any follow-up timepoint (Table 4). There were no differences between groups in serum vitamin D concentration at baseline, but there was a significantly higher change in vitamin D in the ONS+DC group compared with the DC group at 120 d (Table 4). There were no significant differences at baseline (Supplemental Table 5) or in changes at 120 d in vitamin K, A (retinol), B12, E (α-tocopherol), or folate between groups at 120 d (Supplemental Table 7). Cohen’s *d* was calculated for statistically nonsignificant outcomes. A value of ≥0.2 was observed for changes at 120 d for vitamin A (*d* = 0.27), vitamin B12 (*d* = 0.26), and folate (*d* = 0.32).

**TABLE 4:** Eating behaviors and vitamin D over the study period by group^1^

Outcome	Timepoint	ONS+DC		DC		Effect size	*P* value	Adjusted *P* value
		*n*	LSM ± SE	*n*	LSM ± SE	Group difference (DC − ONS+DC; LSM ± SEM)		
Dietary diversity (0–8)	Baseline	173	5.2 ± 0.1	105	5.1 ± 0.2			
	Overall^2^	413^3^	5.71 ± 0.09	268^3^	5.65 ± 0.10	−0.07 ± 0.11	0.53754	
	Day 30^2^	149	5.56 ± 0.11	91	5.40 ± 0.13	−0.16 ± 0.15	0.3039	0.9116
	Day 60^2^	129	5.68 ± 0.11	85	5.58 ± 0.13	−0.10 ± 0.16	0.5149	1.0000
	Day 120^2^	135	5.90 ± 0.12	92	5.97 ± 0.14	0.06 ± 0.16	0.6989	1.0000
Appetite over past 24 h (0 = ate very little; 10 = ate a lot)	Baseline	172	5.6 ± 0.2	104	6.0 ± 0.3			
	Overall^2^	414^3^	6.79 ± 0.14	263^3^	6.73 ± 0.17	−0.06 ± 0.18	0.7342	
	Day 30^2^	148	6.48 ± 0.17	90	6.56 ± 0.21	0.08 ± 0.23	0.7226	1.0000
	Day 60^2^	131	6.78 ± 0.18	83	6.61 ± 0.22	−0.17 ± 0.26	0.5016	1.0000
	Day 120^2^	135	7.11 ± 0.18	90	7.01 ± 0.21	−0.09 ± 0.24	0.6957	1.0000
Vitamin D (ng/mL)	Baseline	139	32.7 ± 1.4	79	35.0 ± 1.7			
	Δ Day 120^4^	87	3.70 ± 1.29	51	0.57 ± 1.43	−3.13 ± 1.44	0.0317^5^	0.0317^5^

Abbreviations: Δ, change from baseline; DC, dietary counseling; LSM, least squares mean; ONS+DC, oral nutrition supplement + dietary counseling.

1 Intent-to-treat data are presented as LSM ± SEM.

2 Timepoints over the intervention were analyzed by repeated measures analysis of covariance (ANCOVA) (study center, gender, study group, study group∗gender, visit, study group∗visit, age group, study group∗age group) with respective baseline value as covariate.

3 The *n* is the sum of all participants at 30, 60, and 120 d.

4 Analyzed by ANCOVA (study group, study center, age group, age group∗study group, gender, gender∗study group) with baseline value as covariate.

5 *P* values indicate significance (*P* < 0.05).

### Activity, sleep, handgrip strength, and questionnaires

For brevity, the following supportive outcome data results are provided in Supplemental Tables 5–10. Baseline supportive characteristics are shown in Supplemental Table 5. There were no significant differences between groups over the study period for handgrip strength, caregiver stress, sleep characteristics, or physical activity levels (Supplemental Table 6). In the PE cohort, however, there was a significantly higher average handgrip strength at baseline in the ONS+DC group compared with the DC group (ONS+DC: 8.1 ± 0.6 kg, *n* = 88 compared with DC: 6.9 ± 0.6 kg, *n* = 80, *P* = 0.0389). There were no differences between groups during the intervention period at days 60 and 120 in CEBQ subscales except for a significant main effect of group on the satiety responsiveness subscale, with the ONS+DC group being significantly more responsive than the DC group, but no individual timepoints were significantly different (Supplemental Table 7). However, the PE cohort showed no main effect of group in CEBQ satiety responsiveness between groups (DC: 3.32 ± 0.07, ONS+DC: 3.19 ± 0.07, *P* = 0.0958). Additionally, in the PE cohort, there was a significantly higher (rather than no difference) change from baseline for CEBQ slowness in eating subscale in the ONS+DC group compared with the DC group (DC: 3.2 ± 0.1, ONS+DC: 3.6 ± 0.1, *P* = 0.0072).

There was a significantly higher incidence (ONS+DC: 29.3% compared with DC: 14.6%; *P* = 0.0074) and number of days [median number of days (interquartile range); ONS+DC: 0.00 (0.00, 1.00) compared with DC: 0.00 (0.00, 0.00), *P* = 0.0129] of missed daycare/group care because of illness in the ONS+DC group compared with the DC group at 120 d (Supplemental Table 8). These differences were likely driven by the higher number of daycare attenders in the ONS+DC group compared with the DC group at baseline. Moreover, no significant differences in these outcomes were seen at baseline or over the study period in the PE cohort. There were no significant differences between groups over the study period in the remaining clinical-related outcomes (Supplemental Tables 8 and 9). No differences were seen between groups in caregiver satisfaction of their child’s growth or confidence in the right nutrition for their child (Supplemental Table 10). Of note, the satisfaction questions were specific to the intervention the caregiver’s child received (i.e., “the recommended nutrition changes” or “the study products and nutrition changes”). There was a significantly higher percentage of caregivers likely to recommend the intervention in which their child received in the ONS+DC group compared with the DC group at 120 d (Very unlikely: DC = 1.3%, ONS+DC = 0.0%, Somewhat unlikely: DC = 1.3%, ONS+DC = 0.0%, Neither: DC = 11.3%, ONS+DC = 2.3%, Somewhat likely: DC = 26.3%, ONS+DC = 18.8%, Very likely: DC = 60.0%, ONS+DC = 78.9%; *P* = 0.0029, Supplementary Table 10). The PE cohort did have a significantly higher percentage (rather than no difference) of caregivers satisfied with their child’s growth in the ONS+DC group compared with the DC group at 120 d (Very unsatisfied: DC: 0.0%, ONS+DC: 1.2%, Somewhat unsatisfied: DC: 2.8%, ONS+DC: 2.3%, Neither: DC: 11.1%, ONS+DC: 3.5%, Somewhat satisfied: DC: 37.5%, ONS+DC: 19.8%, Very satisfied: DC: 48.6%, ONS+DC: 73.3%; *P =* 0.006).

Cohen’s *d* was calculated for statistically nonsignificant outcomes. A value of ≥0.2 was observed for the following variables at the following timepoints: *a*) average daily minutes of activity over the past 7 d at 60 d (wrist accelerometer, *d* = 0.25); *b*) calculated child’s usual amount of sleep per night in hours at 120 d (*d* = 0.24); *c*) calculated child’s usual amount of sleep during the day in hours at 30 d (*d* = 0.21) and 120 d (*d* = 0.21); *d*) calculated child’s usual amount of sleep during a 24-h period in hours at 120 d (*d* = 0.37); *e*) number of times child wakes during the night at day 30 (*d* = 0.37); *f*) CEBQ emotional under-eating score at day 120 (*d* = 0.21); and *g*) CEBQ Satiety responsiveness score at 60 d (*d* = 0.32).

### Adverse events

Overall, there were no safety concerns with the study product (ONS). There were 167 total AEs reported for 99 (35.6%) participants during the study. There were no significant differences between the proportion of participants with ≥1 AE between groups (ONS+DC: 39.3% compared with DC: 29.5%, *P* = 0.10). Fifty-four NSAEs were reported for 31 (29.5%) participants in the DC group and 112 NSAEs reported for 68 (39.3%) participants in the ONS+DC group, a gap of ∼10%. Additionally, there were no statistically significant differences between the groups for any system organ class or preferred term (PT; all *P* values > 0.05), with the exception of the PT influenza (DC: 0% compared with ONS+DC: 4.6%; *P* = 0.03). All reports of the PT influenza were deemed not related to study product by the study physician. Four AEs (of 167) were deemed probably related or possibly related to the study product, of which 2 NSAEs (constipation and rash) from 2 (3.1%) participants in the ONS+DC group were deemed probably related to the study product, and 2 NSAEs (vomiting) from 2 (3.1%) participants were deemed possibly related to the study product by the study physician. The maximum number of NSAEs reported was 5 from 1 (1.0%) participant in the DC group and 1 (0.6%) participant in the ONS+DC group. There was 1 SAE reported for 1 (0.6%) participant in the ONS+DC group (bacterial pharyngitis with otitis media and dehydration), which was treatment emergent and had a severity rating of severe. This event was deemed to be not related to study product by the study physician, and the participant resumed study product once recovered. No SAEs were reported for any of the participants in the DC group. No deaths or other significant AEs were reported.

## Discussion

Pediatric undernutrition is present in the United States population with 1 in 8 children exhibiting ≥1 indicator of undernutrition [16]. More research is needed to evaluate the impacts of supplemental ONS on growth, activity, and behavior in higher-income countries. After 120 d, ONS+DC compared with DC alone showed the following: *1*) significant greater increases from baseline in weight, a standardized height measure, BMI, and MUAC-related growth measures; *2*) no negative influences in parent-perceived childhood appetite; and *3*) significantly greater changes in AMA without excessive fat accumulation. Results support the use of 2 servings of supplemental ONS (480 kcal/d) in addition to DC to improve growth measures more than DC alone over 120 d in children in the United States with ≥1 anthropometric indicator of undernutrition.

Catch-up growth is defined as a period of accelerated growth after a period of illness or starvation [36]. Monitoring catch-up growth through a variety of measurements is essential to improve nutritional status and includes weight, height, BMI, and MUAC. Ideally, DC and taking a food-first approach is the initial recommendation by healthcare providers and dietitians to induce catch-up growth in undernourished children [21]. Improving dietary intake and eating behaviors can promote positive lifelong habits in children to prevent relapse. However, DC may not always adequately increase caloric intake in children [37] in a timely manner to help prevent long-term negative consequences of undernutrition. ONS can be a useful and successful tool for supporting catch-up growth with macronutrients and micronutrients at expert-recommended levels. Together, in as early as 30 d, ONS+DC showed significant improvements for more acute growth indicators such as weight, weight-for-height, BMI, and MUAC. ONS+DC had greater improvements in the less sensitive growth measurements of height across 120 d. Because stunting typically results from longer-term nutritional deprivation [36], [38], [39], height represents a chronic growth indicator and is proposed to take a relatively longer time to adapt to improvements in nutritional intake. Given our shorter study time frame of 4 mo, the improvement in chronic growth measurements showed similar positive relationships in the ONS+DC group over both ITT and PE cohorts and through both AN(C)OVA analyses and repeated measures ANCOVA analyses. These results show that ONS can help support chronic growth indicators in addition to acute growth indicators in a 4-mo timeframe.

The study findings are supported by a plethora of global research showing positive associations of ONS in children with undernutrition [22], and is one of the first to be completed on a United States-only population. To our knowledge, only 2 previous trials on supplemental pediatric ONS consumption have been conducted in the United States [23], [24]. Han et al. [24] showed no effect of ONS consumption, compared with no nutrition intervention, on growth after 6 mo in boys with malnutrition and constitutional growth delay. Differences between our trial and the trial by Han et al. include the inclusion criteria of chronic granulomatous disease (CDGM) in participants and lower ONS energy dose in the Han trial. The authors state that children with CDGM exhibit symptoms similar to children with malnutrition but may have a higher rate of energy expenditure. Given the increased energy needs and fewer calories provided by ONS, this could explain why no differences were seen between groups at 6 mo. Frument et al. [23] showed a positive association of nutritional supplementation and changes in growth outcomes over a 6-mo period, particularly in height when comparing the effect of supplemental ONS compared with usual diet in children. Although results are similar to the trial presented here, some differences observed between our trial and Frument’s may be explained by eligibility criteria. Frument et al. [23] included a wider age range of children (i.e., 4–17 y of age) introducing variability. It is important to note that in both mentioned United States studies, the control groups did not undergo DC to support improvements in nutritional intake in the absence of supplemental ONS. Supplemental ONS are a useful tool to aid in increasing nutritional intake when table foods alone are not enough but are not intended to replace whole food intake. Our study contributes additional data from children living in the United States to the research conducted globally on ONS and children with undernutrition.

Healthy eating habits developed during childhood are long-lasting and impact food intake and dietary behaviors well beyond childhood [40]. The AAP recommends consuming additional protein, micronutrients, and energy to support catch-up growth [13]. As such, DC is the initial recommendation to address undernutrition. However, when food alone is not enough, supplemental ONS may provide a simple and effective option to increase nutritional and caloric intake. A common concern with ONS is that the energy from ONS may impact dietary intake of table foods, potentially affecting appetite or overall dietary patterns. Here, we show that the incorporation of 2 supplemental ONS per day did not impact dietary diversity or appetite over 120 d. These data align with a study showing no change or improved whole food intake in Filipino children when consuming ONS [41], further supporting supplemental ONS as an effective tool in the nutritional management of pediatric undernutrition. Although we did not assess dietary quality or energy, measures of appetite and dietary diversity remained consistent between groups, suggesting that supplemental ONS did not have an adverse effect on intake.

Although pediatric undernutrition is present in the United States, pediatric obesity is at a historic high with ∼1 in 5 children having obesity [42]. DC in children with undernutrition emphasizes consumption of nutrient-dense foods with relatively higher calories to increase energy intake (e.g., avocados, full-fat dairy, addition of oils and/or butter) [43]. There is concern that the incorporation of supplemental ONS into a child’s diet may contribute to overweight and obesity in children who use them, especially in children without a medical indication for the ONS [44]. Our results showed that although mean BMI percentiles increased in the ONS+DC group from ∼10% to 30%, they did not approach the overweight (85th percentile) or obesity (95th percentile) classifications. Participants also did not reach rates of rapid weight gain that have been suggested to be associated with overweight/obesity in childhood [45]. This increase suggests that ONS supported growth without inducing overweight or obesity over a 4-mo period in children with undernutrition. Furthermore, our results also showed that children in the ONS+DC group had a greater increase in mean AMA compared with the DC group alone at 120 d without any differences between groups in AFA or AFI. AMA, AFI, and AFA can be used as proxies for total body muscle and fat [46], [47]. Our results are in line with previous studies utilizing these measures in children who are undernourished [48], including those that utilized dual-energy x-ray absorptiometry measurements [49]. Extrapolating these findings to the whole body would suggest that ONS helped muscle growth without contributing to excess fat gain during growth in undernourished children.

Other outcomes assessed in this trial included handgrip strength, physical activity, sleep, parent-reported health questionnaires, and selected blood serum micronutrient levels. We did not find differences between groups in handgrip strength, parent-reported and wearable measurements of physical activity or sleep, or parent-reported health questionnaires. Our study did find a significantly higher increase in serum vitamin D levels in the ONS+DC group than the DC group at 120 d. There were no differences for the other micronutrients measured. It should be noted that intakes of vitamin D in children in the United States, regardless of undernutrition status, are low [29]. The incorporation of a consistent amount of vitamin D in supplemental ONS could be responsible for improving vitamin D in this trial. It is well documented that handgrip strength [50], physical activity [51], sleep [51], health outcomes, and micronutrient status [52] are adversely affected in children with undernutrition. It is important to point out that these studies show associations with these outcomes in children with moderate to severe undernutrition. Our study focused on children with mild to moderate undernutrition, which may explain why no differences were seen in these measures. Children included in our study may not have experienced undernutrition long enough or severely enough to induce decrements in these outcomes.

Although the results of this study are encouraging, we recognize limitations with our trial. First, a single 24-h dietary recall at each timepoint during the study was used to capture dietary diversity, which does not allow assessment of day-to-day variability [53]. The aim of the dietary recall in this study was to evaluate the intake of different food groups, rather than micronutrient, macronutrient, or energy intakes. Beyond assessment of intake, children may be undernourished by a variety of problems that could be related to eating problems, as well as factors beyond their control, such as parental social, emotional, and behavioral risks. In our study, we show that ONS is a valuable tool within the toolkit of treating pediatric undernutrition, but it should be considered within the context of the children’s eating behaviors, familial cultural considerations, and parental social, emotional, and behavioral influences. Future trials should consider adding assessments that capture factors associated with undernutrition within the family and perceptions of the caregiver/families on the ONS. Second, we included children without chronic illness, which can be another target population for ONS and a potential area for future research. Additionally, the TSF thickness as a measurement for body composition can be influenced by the individual taking measurements. Each investigative site used the same make and model of calipers to ensure consistency and was trained on measuring skinfold thickness to help minimize site variability. Statistical analyses included an adjustment for the impact of site on outcomes to control for this and other potential differences. Third, we included one prespecified primary outcome, of which the study was powered, and included secondary outcomes for which both adjusted and nonadjusted *P* values were reported. Utilizing only the nonadjusted *P* values on secondary outcomes may have inflated the risk of type 1 error (false positives). However, the study results of the secondary outcomes showed internal consistency among the significant differences observed, which suggests that the findings are unlikely to be driven by random variation alone and do not reflect a meaningful risk of false positives. Nonsignificant secondary results should not necessarily be interpreted as evidence of no meaningful difference between groups, as clinically relevant effects may not have been detected because of limited statistical power. To this end, we highlighted those results with a Cohen’s *d* of ≥0.2 (more than a small effect). Fourth, at the end of our trial, we did not have follow-up contact with participants. Cessation of the trial also included cessation of study product, and participants and caregivers would have had to follow-up with their primary care provider on the best next steps for their child. In this study, however, both groups received DC consistent with USDA dietary recommendations and the specific site’s standard of practice by the dietitian. The goal of this counseling is to educate children and their families on the nutritional intake of foods in line with their specific goals. Because of the individual focus on the DC, a further limitation of this design is the variation in DC across sites. Although evaluating the impact of stopping ONS or specific DC practices were not the scope of this trial, future research could evaluate the impact of cessation of ONS on the same growth outcomes and/or the feasibility of a family to continue to purchase the study product or incorporate specified DC recommendations.

This large, multicenter trial contributes to the limited evidence from the United States regarding supplemental ONS consumption in children with undernutrition. Results from our study expand the existing evidence that ONS is a successful option for children with undernutrition already demonstrated in other countries. Given that compliance was high and growth indicators improved, consuming 2 supplemental ONS per day in addition to DC appears to be a feasible and effective recommendation for children behind in growth. Future trials should employ multiple dietary recalls or 3-d diet records at each timepoint to investigate children’s dietary intake with and without supplemental ONS to evaluate the impact of supplemental ONS on macronutrients, micronutrients, and total energy intake and use total body evaluations of lean body mass and fat mass to assess body composition.

## Author contributions

The authors’ responsibilities were as follows – JAW: designed the research; GMN, JAW, MY, GEB: reviewed the data; MY, GEB: analyzed the data; GMN, TLH: drafted the manuscript; PDH, MY, GEB, JAW: critically reviewed the manuscript; GMN: had primary responsibility for final content; and all authors: read and approved the final manuscript.

## Data availability

Pertinent data have been provided in the manuscript, tables, and figures. Additional data described in the manuscript, code book, and analytic code will be made available upon request pending application and approval.

## Declaration of generative AI and AI-assisted technologies in the writing process

The authors declare that no generative AI or AI-assisted technologies were used in the writing of this manuscript.

## Funding

This study was sponsored by Abbott Laboratories, Nutrition Division.

## Conflict of interest

All authors were current employees with Abbott Laboratories, Nutrition Division at the time of study completion, but had no role in data collection.
