# Supplementary material for: Combination of Oral Nutritional Supplements and Dietary Counseling Is More Effective than Dietary Counseling Alone in Improving Growth Indicators among Undernourished United States Children: A Randomized Controlled Clinical Trial
Source: Curr Dev Nutr. 2026 Oct 9;10(11):109552. doi: 10.1016/j.cdnut.2026.109552 (PMC13743383; doi:10.1016/j.cdnut.2026.109552)
Supplement: Supplementary file 1 [file mmc1.docx]

**Supplementary Table 1. Study outcomes and Endpoints**

| **Objectives** | **Endpoints** |
| --- | --- |
| **Primary** | |
| To compare weight-for-age z-score (WAZ) between the intervention and control groups | - Change in WAZ from baseline to 120 days |
| **Secondary** | |
| To compare other growth indicators between the intervention and the control group | - Change in WAZ from baseline to 30 and 60 days - Change in height-for-age *z*-score (HAZ) from baseline to 30, 60 and 120 days - Changes in weight in kg, height in cm, weight-for-age percentile (WAP), height-for-age percentile (HAP), weight-for-height *z*-score (WHZ), weight-for-height percentile (WHP), BMI-for-age *z*-score (BMIAZ), BMI-for-age percentile (BMIAP), and mid-upper-arm circumference-for-age *z*-score (MUACAZ) from baseline to 30, 60 and 120 days |
| To compare dietary intake and appetite between the intervention and control groups | - Dietary diversity scores at baseline, 30, 60 and 120 days - Appetite scores at baseline, 30, 60 and 120 days measured by VAS |
| **Supportive** | |
| To compare hand grip strength between the intervention and control groups | - Hand grip strength in kg at baseline, 60 and 120 days |
| To compare body composition outcomes between the intervention and control groups | - Change in arm muscle area (AMA), arm fat area (AFA) and arm fat index (AFI) from baseline to 30, 60 and 120 days |
| To compare nutrition blood biomarkers between the intervention and control groups | - Changes in serum 25-hydroxyvitamin D, vitamin K1, vitamin A, vitamin B12, vitamin E, and folate from baseline to 120 days |
| To compare clinical-related outcomes outcomes between the intervention and control groups | - Incidences of acute illnesses (number of sick episodes) including gastrointestinal tract infections, and upper and lower respiratory tract infections over the study period as recorded by AE reporting - Number of sick days as recorded by AE reporting and number of days using antibiotics over the study period |
| To compare activity and sleep data between the intervention and control groups | - Average number of hours slept and average minutes of daily activity over the past 7-day period at 30, 60 and 120 days collected from the wrist accelerometer |
| To compare physical activity between the intervention and control groups | - Physical activity levels at baseline, 30, 60 and 120 days measured by VAS |
| To compare caregiver stress between the intervention and control groups | - Caregiver stress levels at baseline, 60 and 120 days |
| To compare child eating behaviors between the intervention and control groups | - Child eating behavior questionnaire at baseline, 60 and 120 days |
| To assess safety | - Adverse events |
| To compare caregiver satisfaction on child’s growth and health status between the intervention and control groups | - Caregiver satisfaction on child’s growth and health status at 120 days - Children’s sleep habit questionnaire at baseline, 30, 60 and 120 days measured by VAS |

**Supplementary Table 2. Study sites used in the study**

| **State** | **Study Site Name: Location** |
| --- | --- |
| **Alabama** | Midway Medical Clinic: Oneonta, Alabama |
| **Arkansas** | HealthStar Research Glenwood: Glenwood, Arkansas  HealthStar Research, LLC: Hot Springs, Arkansas |
| **California** | L.A. Universal Research Center, Inc.: Los Angeles, California |
| **Florida** | Kissimmee Clinical Research: Kissimmee, Florida  Dade Research Center, LLC: Miami, Florida  D&H National Research Centers: Miami, Florida  Suncoast Research, Assoc., LLC: Miami, Florida |
| **Kentucky** | Springs Medical Research: Owensboro, Kentucky |
| **Louisiana** | Velocity Clinical Research: Covington, Louisiana  Southern Clinical Research: Zachary, Louisiana |
| **Nebraska** | Alivation Research (Primary Care): Lincoln, Nebraska |
| **North Carolina** | Javara Inc.: Winston-Salem, North Carolina |
| **South Carolina** | Tribe Clinical Research LLC.: Greenville, South Carolina |
| **Texas** | Javara, Inc.: Dallas, Texas  Proactive Clinical Research: Edinburg, Texas  Gentle Pediatrics: Sugar Land, Texas |

**Supplementary Table 3. Nutritional Supplement Composition**

|  | **Experimental ONS** | **% DV^a^** |
| --- | --- | --- |
| Serving Size | 8 fl oz |  |
| Calories | 240 |  |
| Total Fat, g | 9 | 12% |
| Sat Fat, g | 1.5 | 8% |
| Trans Fat, g | 0 |  |
| Polyunsaturated fat, g | 3.5 |  |
| Monounsaturated fat, g | 3.5 |  |
| Cholesterol, mg |  |  |
| Vanilla, Strawberry, Banana Flavors | 5 | <2% |
| Chocolate Flavor | 10 | 3% |
| Sodium, mg | 90 | 4% |
| Total Carbohydrate, g | 33 | 12% |
| Dietary Fiber, g | <1 | <4% |
| Vanilla, Strawberry, Banana Flavors | 1 | 4% |
| Chocolate, S’mores Flavors | <1 | <2% |
| Total Sugars, g | 9 |  |
| Added sugars, g | 9 | 18% |
| Protein, g | 7 | 14% |
| Biotin |  | 25% |
| Calcium, mg | 330 | 25% |
| Chloride |  | 10% |
| Choline |  | 15% |
| Chromium |  |  |
| Vanilla, Strawberry, Banana Flavors |  | 25% |
| Chocolate Flavor |  | 35% |
| Copper |  | 15% |
| Folate, mcg Folic Acid | 36 | 15% |
| Iodine |  | 15% |
| Iron, mg | 2.7 | 15% |
| Magnesium |  | 10% |
| Manganese |  | 20% |
| Molybdenum |  | 20% |
| Niacin |  | 20% |
| Pantothenic Acid |  | 25% |
| Phosphorus |  | 20% |
| Potassium, mg | 470 | 10% |
| Riboflavin |  | 25% |
| Selenium |  | 15% |
| Thiamin |  | 25% |
| Vitamin A |  | 15% |
| Vitamin B12 |  | 20% |
| Vitamin B6 |  | 20% |
| Vitamin C |  | 25% |
| Vitamin D, mcg | 6 | 30% |
| Vitamin E |  | 20% |
| Vitamin K |  | 15% |
| Zinc |  | 15% |
| **Ingredients (Vanilla Flavor)**: Water, Corn Maltodextrin, Blend of Vegetable Oils (Soy, High Oleic Safflower), Sugar, Milk Protein Concentrate, Soy Protein Isolate. Less than 0.5% of: Vitamins & Minerals (Potassium Citrate, Calcium Phosphate, Potassium Chloride, Magnesium Phosphate, Potassium Phosphate, Calcium Carbonate, Ascorbic Acid, Choline Chloride, Ferrous Sulfate, dl-Alpha-Tocopheryl Acetate, Zinc Sulfate, Niacinamide, Calcium Pantothenate, Manganese Sulfate, Thiamine Hydrochloride, Pyridoxine Hydrochloride, Riboflavin, Vitamin A Palmitate, Copper Sulfate, Folic Acid, Chromium Chloride, Potassium Iodide, Sodium Molybdate, Sodium Selenate, Biotin, Phylloquinone, Vitamin D3, Vitamin B12, Menaquinone-7), Natural & Artificial Flavors, Cellulose Gel, Tuna Oil, Cellulose Gum, Monoglycerides, Soy Lecithin, Carrageenan, Potassium Hydroxide, Inositol, Salt, Taurine, Stevia Leaf Extract, L-Carnitine, and Lutein. | | |
| **^a^Percent Daily Value is based on the Daily Value for individuals 4 years and older.** | | |

**Supplementary Table 4. Participant characteristics at study entry by group^a^**

|  | **ONS+DC** | **DC** |
| --- | --- | --- |
| **Birth Weight (g)** |  |  |
| **LSM ± SEM** | 3031.8 ± 66.4 | 2977.6 ± 77.7 |
| **n** | 171 | 105 |
| **Birth Length (computed cm)** |  |  |
| **LSM ± SEM** | 48.3 ± 0.7 | 48.4 ± 0.8 |
| **n** | 167 | 103 |
| **Gestational Age (weeks)** |  |  |
| **LSM ± SEM** | 38.2 ± 0.3 | 37.8 ± 0.3 |
| **n** | 172 | 105 |
| **Birth Status, n (%)** |  |  |
| **Premature** | 34 (19.8) | 15 (14.3) |
| **Term** | 138 (80.2) | 90 (85.7) |
| **n** | 172 | 105 |
| **Birth Order, n (%)** |  |  |
| **1** | 46 (32.2) | 15 (17.0) |
| **2** | 54 (37.8) | 42 (47.7) |
| **3** | 25 (17.5) | 17 (19.3) |
| **4** | 8 (5.6) | 9 (10.2) |
| **5** | 5 (3.5) | 3 (3.4) |
| **6** | 2 (1.4) | 1 (1.1) |
| **7** | 3 (2.1) | 1 (1.1) |
| **Mothers Age (years)** |  |  |
| **LSM ± SEM** | 34.1 ± 0.6 | 34.8 ± 0.7 |
| **n** | 172 | 104 |
| **Fathers Age (years)** |  |  |
| **LSM ± SEM** | 37.8 ± 0.9 | 37.7 ± 1.0 |
| **n** | 165 | 105 |
| **Mothers Weight (lbs)** |  |  |
| **LSM ± SEM** | 163.3 ± 3.8 | 165.8 ± 4.4 |
| **n** | 172 | 104 |
| **Fathers Weight (lbs)** |  |  |
| **LSM ± SEM** | 185.5 ± 4.0 | 190.8 ± 4.6 |
| **n** | 164 | 102 |
| **Number of Kids Under 18 in Household** |  |  |
| **LSM ± SEM** | 2.6 ± 0.1 | 2.6 ± 0.2 |
| **n** | 173 | 105 |
| **Current Marital Status, n (%)** |  |  |
| **Never married** | 49 (28.3) | 30 (28.6) |
| **Currently married** | 102 (59.0) | 65 (61.9) |
| **Separated** | 10 (5.8) | 7 (6.7) |
| **Divorced** | 11 (6.4) | 3 (2.9) |
| **Widowed** | 1 (0.6) | 0 (0.0) |
| **Mothers Highest Level of Education, n (%)** |  |  |
| **Postgraduate Degree or Doctoral** | 9 (5.2) | 6 (5.7) |
| **College or University Degree Complete** | 48 (27.7) | 27 (25.7) |
| **Associate or Technical Degree Complete** | 31 (17.9) | 17 (16.2) |
| **High School Diploma** | 67 (38.7) | 41 (39.0) |
| **Secondary School** | 9 (5.2) | 6 (5.7) |
| **Primary School or less** | 5 (2.9) | 4 (3.8) |
| **Refused** | 4 (2.3) | 4 (3.8) |
| **Fathers Highest Level of Education , n (%)** |  |  |
| **Postgraduate Degree or Doctoral** | 13 (7.6) | 8 (7.6) |
| **College or University Degree Complete** | 30 (17.4) | 24 (22.9) |
| **Associate or Technical Degree Complete** | 36 (20.9) | 14 (13.3) |
| **High School Diploma** | 72 (41.9) | 40 (38.1) |
| **Secondary School** | 5 (2.9) | 4 (3.8) |
| **Primary School or less** | 6 (3.5) | 5 (4.8) |
| **Refused** | 10 (5.8) | 10(9.5) |
| **Mothers Employment Status, n (%)** |  |  |
| **Working - Full-time** | 66 (38.2) | 41 (39.0) |
| **Working - part-time** | 27 (15.6) | 18 (17.1) |
| **Homemaker / Housewife** | 57 (32.9) | 30 (28.6) |
| **Unemployed (able to work)** | 2 (1.2) | 2 (1.9) |
| **Unemployed (unable to work)** | 5 (2.9) | 2 (1.9) |
| **Refused** | 16 (9.2) | 12 (11.4) |
| **Fathers Employment Status, n (%)** |  |  |
| **Working - Full-time** | 115 (67.3) | 75 (71.4) |
| **Working - part-time** | 11 (6.4) | 4 (3.8) |
| **Homemaker / Housewife** | 2 (1.2) | 1 (1.0) |
| **Retired** | 2 (1.2) | 0 (0.0) |
| **Unemployed (able to work)** | 2 (1.2) | 2 (1.9) |
| **Unemployed (unable to work)** | 5 (2.9) | 1 (1.0) |
| **Refused** | 34 (19.9) | 22 (21.0) |
| ^a^Intent-to-treat data are presented as Least Squares Mean (LSM) ± Standard Error of the Mean (SEM) or number of respondents | | |

**Supplementary Table 5: Baseline Eating Behaviors and Micronutrients^a^**

|  | **ONS ± DC** | | **DC** | |
| --- | --- | --- | --- | --- |
|  | **n** | **LSM±SEM** | **n** | **LSM±SEM** |
| **Caregiver Stress Score** | 173 | 32.2 ± 0.9 | 105 | 33.1 ± 1.0 |
| **Child’s Usual Amount of Sleep Per Night [Hours, calculated]**  **Median (Interquartile Range)** | 162 | 10.00 (9.00, 10.50) | 97 | 10.00 (9.00, 10.50) |
| **Child’s Usual Amount of Sleep During the Day [Hours, calculated]**  **Median (Interquartile Range)** | 164 | 0.00 (0.00, 1.00) | 101 | 0.00 (0.00, 1.00) |
| **Child’s Usual Amount of Sleep During a 24-hr period [Hours, calculated]**  **Median (Interquartile Range)** | 160 | 10.08 (9.00, 11.00) | 96 | 10.00 (9.13, 11.00) |
| **Number of Times Child Wakes During the Night; Median (Interquartile Range)** | 172 | 0.00 (0.00, 1.00) | 105 | 0.00 (0.00, 1.00) |
| **Child’s Activity Level Over the Past 24 Hours (0=Not Active, 10=Very Active)** | 172 | 8.3 ± 0.2 | 103 | 8.2 ± 0.2 |
| **Handgrip Strength (kg)** | 170 | 7.5 ± 0.4 | 102 | 6.9 ± 0.4 |
| **Food Responsiveness CEBQ** | 173 | 2.2 ± 0.1 | 105 | 2.4 ± 0.1 |
| **Emotional Over-Eating CEBQ** | 173 | 1.6 ± 0.1 | 105 | 1.7 ± 0.1 |
| **Slowness in Eating CEBQ** | 173 | 3.4 ± 0.1 | 105 | 3.2 ± 0.1 |
| **Emotional Under-Eating CEBQ** | 173 | 2.8 ± 0.1 | 105 | 2.6 ± 0.1 |
| **Enjoyment of Food CEBQ** | 173 | 3.2 ± 0.1 | 105 | 3.2 ± 0.1 |
| **Food Fussiness CEBQ** | 173 | 3.4 ± 0.1 | 105 | 3.3 ± 0.1 |
| **Desire to Drink CEBQ** | 173 | 3.1 ± 0.1 | 105 | 3.1 ± 0.1 |
| **Satiety Responsiveness CEBQ** | 173 | 3.5 ± 0.1 | 105 | 3.4 ± 0.1 |
| **Alpha-tocopherol (mg/dL)** | 136 | 9.8 ± 0.4 | 80 | 10.2 ± 0.4 |
| **Folate (ng/mL)^b^** | 101 | 20.7 ± 1.3 | 65 | 21.4 ± 1.3 |
| **Retinol (μg/dL)** | 134 | 33.4 ± 1.1 | 80 | 35.0 ± 1.3 |
| **Vitamin B12 (pg/mL)** | 116 | 789.4 ± 60.3 | 66 | 699.0 ± 69.4 |
| **Vitamin D (ng/mL)** | 139 | 32.7 ± 1.4 | 79 | 35.0 ± 1.7 |
| **Vitamin K (pg/mL)^c^** | 126 | 433.5 ± 58. | 75 | 334.2 ± 66.1 |
| ^a^Intent-to-treat data are presented as Least Squares Mean (LSM) ± Standard Error of the Mean (SEM); ^b^Values > 48 ng/mL were defined as 48 ng/mL; ^c^Values < 50 pg/mL were defined as 25 pg/mL, and values > 2500 pg/mL were defined as 2500 pg/mL; CEBQ: Childhood Eating Behavior Questionnaire; DC: dietary counseling; ONS+DC: oral nutrition supplement + dietary counseling | | | | |

**Supplementary Table 6. Physical Activity, Sleep, and Strength over the study period by group^a^**

| **Outcome** |  | **ONS+DC** | | **DC** | | ***P*-value** |
| --- | --- | --- | --- | --- | --- | --- |
|  |  | **n** | **LSM±SEM** | **n** | **LSM±SEM** |  |
| **Average Number of Hours Child Slept over the Past 7 Days (Wrist Accelerometer)^b^** | **Overall** | 361^d^ | 9.57 ± 0.17 | 230^d^ | 9.35 ± 0.19 | 0.25 |
|  | **Day 30** | 135 | 9.49 ± 0.19 | 83 | 9.27 ± 0.21 | 0.93 |
|  | **Day 60** | 116 | 9.63 ± 0.20 | 76 | 9.37 ± 0.23 | 0.93 |
|  | **Day 120** | 110 | 9.59 ± 0.20 | 71 | 9.40 ± 0.22 | 0.93 |
| **Average Daily Minutes of Child’s Activity Over the Past 7 Days (Wrist Accelerometer)^b^** | **Overall** | 338^d^ | 76.02 ± 5.34 | 215^d^ | 81.44 ± 5.85 | 0.37 |
|  | **Day 30** | 126 | 78.49 ± 6.22 | 78 | 80.47 ± 7.15 | 1.00 |
|  | **Day 60** | 109 | 68.87 ± 6.06 | 72 | 82.70 ± 6.76 | 0.20 |
|  | **Day 120** | 103 | 80.69 ± 6.23 | 65 | 81.16 ± 7.12 | 1.00 |
| **Child’s Activity Level over the past 24 hours (0=not active, 10=very active)^c^** | **Overall** | 414^d^ | 8.60 ± 0.11 | 261^d^ | 8.52 ± 0.13 | 0.58 |
|  | **Day 30** | 149 | 8.44 ± 0.14 | 89 | 8.39 ± 0.17 | 1.00 |
|  | **Day 60** | 131 | 8.65 ± 0.13 | 83 | 8.46 ± 0.15 | 0.84 |
|  | **Day 120** | 134 | 8.69 ± 0.15 | 89 | 8.70 ± 0.17 | 1.00 |
| **Child’s Overall Sleep Quality (0=Very Poor, 10=Very Good)^c^** | **Overall** | 409^d^ | 8.77 ± 0.09 | 263^d^ | 8.73 ± 0.11 | 0.76 |
|  | **Day 30** | 147 | 8.59 ± 0.12 | 90 | 8.70 ± 0.14 | 1.00 |
|  | **Day 60** | 131 | 8.77 ± 0.11 | 83 | 8.69 ± 0.13 | 1.00 |
|  | **Day 120** | 131 | 8.95 ± 0.12 | 90 | 8.80 ± 0.14 | 1.00 |
| **Child’s Usual Amount of Sleep Per Night (Hours, calculated)^c^** | **Overall** | 385^d^ | 9.67 ± 0.09 | 241^d^ | 9.62 ± 0.10 | 0.69 |
|  | **Day 30** | 139 | 9.58 ± 0.10 | 83 | 9.58 ± 0.12 | 0.99 |
|  | **Day 60** | 122 | 9.71 ± 0.11 | 76 | 9.83 ± 0.13 | 0.91 |
|  | **Day 120** | 124 | 9.71 ± 0.13 | 82 | 9.46 ± 0.15 | 0.50 |
| **Child’s Usual Amount of Sleep During the Day (Hours, calculated)^c^** | **Overall** | 388^d^ | 0.62 ± 0.06 | 246^d^ | 0.50 ± 0.07 | 0.14 |
|  | **Day 30** | 138 | 0.69 ± 0.09 | 86 | 0.52 ± 0.11 | 0.65 |
|  | **Day 60** | 124 | 0.55 ± 0.07 | 76 | 0.48 ± 0.08 | 0.65 |
|  | **Day 120** | 126 | 0.62 ± 0.08 | 84 | 0.50 ± 0.10 | 0.65 |
| **Child’s Usual Amount of Sleep During a 24-hr period (Hours, calculated)^c^** | **Overall** | 376^d^ | 10.28 ± 0.10 | 234^d^ | 10.12 ± 0.12 | 0.23 |
|  | **Day 30** | 134 | 10.23 ± 0.11 | 81 | 10.10 ± 0.14 | 0.83 |
|  | **Day 60** | 120 | 10.27 ± 0.12 | 73 | 10.32 ± 0.14 | 0.83 |
|  | **Day 120** | 122 | 10.35 ± 0.15 | 80 | 9.95 ± 0.18 | 0.21 |
| **Number of Times Child Wakes During the Night^b^** | **Overall** | 409^d^ | 0.67 ± 0.09 | 261^d^ | 0.77 ± 0.10 | 0.39 |
|  | **Day 30** | 149 | 0.74 ± 0.18 | 90 | 1.05 ± 0.23 | 0.80 |
|  | **Day 60** | 129 | 0.62 ± 0.07 | 83 | 0.61 ± 0.08 | 1.00 |
|  | **Day 120** | 132 | 0.65 ± 0.09 | 91 | 0.66 ± 0.10 | 1.00 |
| **Caregiver Stress Score^c^** | **Overall** | 263^d^ | 30.05 ± 0.61 | 176^d^ | 30.60 ± 0.68 | 0.45 |
|  | **Day 60** | 128 | 29.98 ± 0.62 | 84 | 30.74 ± 0.70 | 0.62 |
|  | **Day 120** | 135 | 30.13 ± 0.69 | 92 | 30.46 ± 0.77 | 0.70 |
| **Average Handgrip Strength (kg)^c^** | **Overall** | 261^d^ | 7.04 ± 0.23 | 171^d^ | 6.84 ± 0.26 | 0.49 |
|  | **Day 60** | 128 | 6.71 ± 0.22 | 81 | 6.62 ± 0.25 | 0.87 |
|  | **Day 120** | 133 | 7.36 ± 0.28 | 90 | 7.07 ± 0.32 | 0.87 |
| **Change in Handgrip Strength (kg)^c^** | **Overall** | 261^d^ | 0.58 ± 0.23 | 171^d^ | 0.39 ± 0.26 | 0.49 |
|  | **ΔDay 60** | 128 | 0.26 ± 0.22 | 81 | 0.16 ± 0.25 | 0.87 |
|  | **ΔDay 120** | 133 | 0.91 ± 0.28 | 90 | 0.61 ± 0.32 | 0.87 |
| ^a^Intent-to-treat data are presented as Least Squares Mean (LSM) ± Standard Error of the Mean (SEM); ^b^Data were analyzed by repeated measures ANOVA (Study center, gender, study group, study group*gender, visit, study group*visit, age group, study group*age group); ^c^Data were analyzed by repeated measures ANCOVA (Study center, gender, study group, study group*gender, visit, study group*visit, age group, study group*age group) with respective baseline value as covariate; ^d^The n is the sum of all participants at all timepoints; DC: dietary counseling; ONS+DC: oral nutrition supplement + dietary counseling; bolded *P*-values indicate significance | | | | | | |

**Supplementary Table 7. Eating Behaviors and Micronutrients Over the Study Period by Group^a^**

| **Outcome** |  | **ONS+DC** | | **DC** | | **Effect Size** | ***P*-value** |
| --- | --- | --- | --- | --- | --- | --- | --- |
|  |  | **n** | **LSM±SE** | **n** | **LSM±SE** | **Group Difference (DC – ONS+DC; LSM±SEM)** |  |
| **Food Responsiveness CEBQ^b^** | **Overall** | 261^d^ | 2.34 ± 0.06 | 176^d^ | 2.30 ± 0.07 | -0.04 ± 0.07 | 0.56 |
|  | **Day 60** | 126 | 2.29 ± 0.06 | 84 | 2.24 ± 0.07 | -0.05 ± 0.08 | 1.00 |
|  | **Day 120** | 135 | 2.40 ± 0.07 | 92 | 2.36 ± 0.07 | -0.04 ± 0.08 | 1.00 |
| **Emotional Over-Eating CEBQ^b^** | **Overall** | 261^d^ | 1.92 ± 0.05 | 176^d^ | 1.85 ± 0.06 | -0.07 ± 0.06 | 0.30 |
|  | **Day 60** | 126 | 1.91 ± 0.06 | 84 | 1.84 ± 0.07 | -0.07 ± 0.07 | 0.69 |
|  | **Day 120** | 135 | 1.93 ± 0.06 | 92 | 1.87 ± 0.07 | -0.06 ± 0.08 | 0.69 |
| **Slowness in Eating CEBQ^b^** | **Overall** | 261^d^ | 3.19 ± 0.06 | 176^d^ | 3.18 ± 0.07 | -0.01 ± 0.07 | 0.92 |
|  | **Day 60** | 126 | 3.29 ± 0.07 | 84 | 3.19 ± 0.07 | -0.11 ± 0.08 | 0.40 |
|  | **Day 120** | 135 | 3.08 ± 0.07 | 92 | 3.17 ± 0.08 | 0.09 ± 0.09 | 0.40 |
| **Emotional Under-Eating CEBQ^b^** | **Overall** | 261^d^ | 3.00 ± 0.07 | 176^d^ | 2.89 ± 0.08 | -0.11 ± 0.086 | 0.20 |
|  | **Day 60** | 126 | 3.04 ± 0.08 | 84 | 2.88 ± 0.09 | -0.16 ± 0.1 | 0.24 |
|  | **Day 120** | 135 | 2.95 ± 0.08 | 92 | 2.89 ± 0.09 | -0.07 ± 0.10 | 0.52 |
| **Enjoyment of Food CEBQ^b^** | **Overall** | 261^d^ | 3.24 ± 0.06 | 176^d^ | 3.26 ± 0.07 | 0.02 ± 0.07 | 0.84 |
|  | **Day 60** | 126 | 3.19 ± 0.07 | 84 | 3.23 ± 0.08 | 0.04 ± 0.08 | 1.00 |
|  | **Day 120** | 135 | 3.30 ± 0.07 | 92 | 3.29 ± 0.08 | -0.01 ± 0.09 | 1.00 |
| **Food Fussiness CEBQ^b^** | **Overall** | 261^d^ | 3.26 ± 0.06 | 176^d^ | 3.31 ± 0.06 | 0.05 ± 0.07 | 0.42 |
|  | **Day 60** | 126 | 3.28 ± 0.06 | 84 | 3.36 ± 0.07 | 0.08 ± 0.08 | 0.56 |
|  | **Day 120** | 135 | 3.23 ± 0.06 | 92 | 3.26 ± 0.07 | 0.03 ± 0.08 | 0.74 |
| **Desire to Drink CEBQ^b^** | **Overall** | 261^d^ | 3.17 ± 0.08 | 176^d^ | 3.14 ± 0.09 | -0.04 ± 0.09 | 0.70 |
|  | **Day 60** | 126 | 3.22 ± 0.08 | 84 | 3.14 ± 0.10 | -0.08 ± 0.11 | 0.95 |
|  | **Day 120** | 135 | 3.12 ±0 .09 | 92 | 3.13 ± 0.10 | 0.01 ± 0.11 | 0.97 |
| **Satiety Responsiveness CEBQ^b^** | **Overall** | 261^d^ | 3.25 ± 0.05 | 176^d^ | 3.37 ± 0.06 | 0.13 ± 0.06 | **0.04** |
|  | **Day 60** | 126 | 3.29 ± 0.06 | 84 | 3.39 ± 0.07 | 0.1 ± 0.07 | 0.19 |
|  | **Day 120** | 135 | 3.21 ± 0.06 | 92 | 3.36 ± 0.07 | 0.16 ± 0.07 | 0.07 |
| **Vitamin K (pg/mL)^c,e^** | **ΔDay 120** | 80 | 123.51 ± 57.76 | 46 | 73.89 ± 66.82 | -49.62 ± 70.64 | 0.48 |
| **Vitamin A (retinol) (μg/dL)^c^** | **ΔDay 120** | 84 | -2.95 ± 1.22 | 51 | -1.13 ± 1.31 | 1.82 ± 1.34 | 0.18 |
| **Vitamin B12 (pg/mL)^c^** | **ΔDay 120** | 67 | 94.71 ± 63.37 | 39 | 17.76 ± 75.14 | -76.94 ± 69.42 | 0.27 |
| **Vitamin E (α-tocopherol) (mg/L)^c^** | **ΔDay 120** | 85 | -0.50 ± 0.39 | 51 | -0.63 ± 0.41 | -0.13 ± 0.4 | 0.74 |
| **Folate (ng/mL)^c,f^** | **ΔDay 120** | 52 | 2.30 ± 1.52 | 35 | 0.29 ± 1.50 | -2.01 ± 1.56 | 0.20 |
| ^a^Intent-to-treat data are presented as Least Squares Mean (LSM) ± Standard Error of the Mean (SEM); ^b^Analyzed by repeated measures ANCOVA (Study center, gender, study group, study group*gender, visit, study group*visit, age group, study group*age group) with respective baseline value as covariate; ^c^Analyzed by ANCOVA (study group, study center, age group, age group*study group, gender, gender*study group) with baseline value as covariate; ^d^The n is the sum of all participants at all timepoints; ^e^Values > 48 ng/mL were defined as 48 ng/mL; ^f^Values < 50 pg/mL were defined as 25 pg/mL, and values > 2500 pg/mL were defined as 2500 pg/mL; CEBQ: Childhood Eating Behavior Questionnaire; DC: dietary counseling; ONS+DC: oral nutrition supplement + dietary counseling; bolded *P*-values indicate significance | | | | | | | |

**Supplementary Table 8. Clinical Outcomes over the study period by group^a^**

| **Outcome** | | | **ONS+DC** | **DC** | ***P*-value** |
| --- | --- | --- | --- | --- | --- |
|  |  |  | **n (%)** | **n (%)** |  |
| **Was Participant Seen by an HCP for Non-Routine Well Visit (from baseline)?** | **Day 30** | **Yes** | 22 (14.9) | 9 (9.9) | 0.32 |
|  |  | **No** | 126 (85.1) | 82 (90.1) |  |
|  |  | **Total** | 148 (100.0) | 91 (100.0) |  |
|  | **Day 60** | **Yes** | 29 (17.8) | 11 (10.9) | 0.16 |
|  |  | **No** | 134 (82.2) | 90 (89.1) |  |
|  |  | **Total** | 163 (100.0) | 101 (100.0) |  |
|  | **Day 120** | **Yes** | 41 (25.2) | 18 (17.5) | 0.17 |
|  |  | **No** | 122 (74.8) | 85 (82.5) |  |
|  |  | **Total** | 163 (100.0) | 103 (100.0) |  |
| **Urgent Care Visits Since Last Visit (Y/N, from baseline)** | **Day 30** | **Yes** | 5 (3.4) | 2 (2.2) | 0.71 |
|  |  | **No** | 143 (96.6) | 89 (97.8) |  |
|  |  | **Total** | 148 (100.0) | 91 (100.0) |  |
|  | **Day 60** | **Yes** | 7 (4.3) | 2 (2.0) | 0.49 |
|  |  | **No** | 156 (95.7) | 99 (98.0) |  |
|  |  | **Total** | 163 (100.0) | 101 (100.0) |  |
|  | **Day 120** | **Yes** | 10 (6.1) | 3 (2.9) | 0.38 |
|  |  | **No** | 153 (93.9) | 100 (97.1) |  |
|  |  | **Total** | 163 (100.0) | 103 (100.0) |  |
| **Emergency Department Visits Since Last Visit (Y/N; from baseline)** | **Day 30** | **Yes** | 2 (1.4) | 0 (0.0) | 0.53 |
|  |  | **No** | 146 (98.6) | 91 (100.0) |  |
|  |  | **Total** | 148 (100.0) | 91 (100.0) |  |
|  | **Day 60** | **Yes** | 4 (2.5) | 0 (0.0) | 0.30 |
|  |  | **No** | 159 (97.5) | 101 (100.0) |  |
|  |  | **Total** | 163 (100.0) | 101 (100.0) |  |
|  | **Day 120** | **Yes** | 6 (3.7) | 2 (1.9) | 0.49 |
|  |  | **No** | 157 (96.3) | 101 (98.1) |  |
|  |  | **Total** | 163 (100.0) | 103 (100.0) |  |
| **Primary Care HCP/Outpatient Clinical Visits Since Last Visit (Y/N, from baseline)** | **Day 30** | **Yes** | 13 (8.8) | 7 (7.7) | 0.82 |
|  |  | **No** | 135 (91.2) | 84 (92.3) |  |
|  |  | **Total** | 148 (100.0) | 91 (100.0) |  |
|  | **Day 60** | **Yes** | 18 (11.0) | 9 (8.9) | 0.68 |
|  |  | **No** | 145 (89.0) | 92 (91.1) |  |
|  |  | **Total** | 163 (100.0) | 101 (100.0) |  |
|  | **Day 120** | **Yes** | 30 (18.4) | 15 (14.6) | 0.50 |
|  |  | **No** | 133 (81.6) | 88 (85.4) |  |
|  |  | **Total** | 163 (100.0) | 103 (100.0) |  |
| **Specialist Visits Since Last Visit (Y/N, from baseline)** | **Day 30** | **Yes** | 1 (0.7) | 0 (0.0) | 1.00 |
|  |  | **No** | 147 (99.3) | 91 (100.0) |  |
|  |  | **Total** | 148 (100.0) | 91 (100.0) |  |
|  | **Day 60** | **Yes** | 3 (1.8) | 0 (0.0) | 0.29 |
|  |  | **No** | 160 (98.2) | 101 (100.0) |  |
|  |  | **Total** | 163 (100.0) | 101 (100.0) |  |
|  | **Day 120** | **Yes** | 5 (3.1) | 1 (1.0) | 0.41 |
|  |  | **No** | 158 (96.9) | 102 (99.0) |  |
|  |  | **Total** | 163 (100.0) | 103 (100.0) |  |
| **Missed any Daycare/Group Care for Illness (Y/N, from baseline)?** | **Day 30** | **Yes** | 21 (14.1) | 11 (12.1) | 0.70 |
|  |  | **No** | 128 (85.9) | 80 (87.9) |  |
|  |  | **Total** | 149 (100.0) | 91 (100.0) |  |
|  | **Day 60** | **Yes** | 32 (19.5) | 13 (12.9) | 0.18 |
|  |  | **No** | 132 (80.5) | 88 (87.1) |  |
|  |  | **Total** | 164 (100.0) | 101 (100.0) |  |
|  | **Day 120** | **Yes** | 48 (29.3) | 15 (14.6) | **<0.01** |
|  |  | **No** | 116 (70.7) | 88 (85.4) |  |
|  |  | **Total** | 164 (100.0) | 103 (100.0) |  |
| **Did Parent Miss Work for Child’s Illness (Y/N, from baseline)?** | **Day 30** | **Yes** | 7 (4.7) | 5 (5.5) | 0.77 |
|  |  | **No** | 141 (95.3) | 86 (94.5) |  |
|  |  | **Total** | 148 (100.0) | 91 (100.0) |  |
|  | **Day 60** | **Yes** | 11 (6.8) | 5 (5.0) | 0.61 |
|  |  | **No** | 151 (93.2) | 96 (95.0) |  |
|  |  | **Total** | 162 (100.0) | 101 (100.0) |  |
|  | **Day 120** | **Yes** | 18 (11.1) | 8 (7.8) | 0.41 |
|  |  | **No** | 144 (88.9) | 95 (92.2) |  |
|  |  | **Total** | 162 (100.0) | 103 (100.0) |  |
| ^a^Intent-to-treat data are presented as incidence and were analyzed by Fisher’s exact test; HCP: Healthcare Professional; Y=yes, N=no; DC: dietary counseling; ONS+DC: oral nutrition supplement + dietary counseling; bolded *P*-values indicate significance | | | | | |

**Supplementary Table 9. Clinical Outcomes over the study period by group^a^**

| **Outcome** | | **ONS+DC** | | **DC** | | ***P*-value** |
| --- | --- | --- | --- | --- | --- | --- |
|  | | **n** | **Result** | **n** | **Result** |  |
| **Incidence of Acute Illnesses (Including Gastrointestinal Tract Infections, RTI, Ear infections)^b^ , n (%)** | **Yes** |  | 59 (34.1) |  | 26 (24.8) | 0.10 |
|  | **No** |  | 114 (65.9) |  | 79 (75.2) |  |
|  | **Total** |  | 173 (100) |  | 105 (100) |  |
| **Number of AE Reports of Acute Illnesses (Including Gastrointestinal Tract Infections, RTI, Ear infections)^d^**  **Median (Interquartile Range)** |  | 173 | 0.00 (0.00, 1.00) | 105 | 0.00 (0.00, 0.00) | 0.13 |
| **Days of Oral/IV Antibiotic Use by Child Over the Study Period^c^**  **LSM±SEM** |  | 173 | 3.1 ± 0.6 | 105 | 3.8 ± 0.7 | 0.38 |
| **Number of Times Participant was Seen by an HCP for Non-routine Well Visits (From baseline)^d^**  **Median (Interquartile Range)** | **Day 30** | 148 | 0.00 (0.00, 0.00) | 91 | 0.00 (0.00, 0.00) | 0.38 |
|  | **Day 60** | 163 | 0.00 (0.00, 0.00) | 101 | 0.00 (0.00, 0.00) | 0.20 |
|  | **Day 120** | 163 | 0.00 (0.00, 1.00) | 103 | 0.00 (0.00, 0.00) | 0.17 |
| **Number of Days Missed of Daycare/Group Care for Illness (from baseline)^d^**  **Median (Interquartile Range)** | **Day 30** | 149 | 0.00 (0.00, 0.00) | 91 | 0.00 (0.00, 0.00) | 0.61 |
|  | **Day 60** | 163 | 0.00 (0.00, 0.00) | 101 | 0.00 (0.00, 0.00) | 0.18 |
|  | **Day 120** | 163 | 0.00 (0.00, 1.00) | 103 | 0.00 (0.00, 0.00) | 0.01 |
| **Number of Days Parent Missed Work for Child’s Illness (from baseline)^d^**  **Median (Interquartile Range)** | **Day 30** | 148 | 0.00 (0.00, 0.00) | 91 | 0.00 (0.00, 0.00) | 0.83 |
|  | **Day 60** | 162 | 0.00 (0.00, 0.00) | 101 | 0.00 (0.00, 0.00) | 0.51 |
|  | **Day 120** | 162 | 0.00 (0.00, 0.00) | 103 | 0.00 (0.00, 0.00) | 0.33 |
| **Number of Emergency Department Visits (from baseline)^d^**  **Median (Interquartile Range)^d^** | **Day 30** | 148 | 0.00 (0.00, 0.00) | 91 | 0.00 (0.00, 0.00) | 0.27 |
|  | **Day 60** | 163 | 0.00 (0.00, 0.00) | 101 | 0.00 (0.00, 0.00) | 0.11 |
|  | **Day 120** | 163 | 0.00 (0.00, 0.00) | 103 | 0.00 (0.00, 0.00) | 0.42 |
| **Number of Primary Care HCP/Outpatient Clinical Visits (from baseline)^d^**  **Median (Interquartile Range)** | **Day 30** | 148 | 0.00 (0.00, 0.00) | 91 | 0.00 (0.00, 0.00) | 0.76 |
|  | **Day 60** | 163 | 0.00 (0.00, 0.00) | 101 | 0.00 (0.00, 0.00) | 0.57 |
|  | **Day 120** | 163 | 0.00 (0.00, 0.00) | 103 | 0.00 (0.00, 0.00) | 0.44 |
| **Number of Urgent Care Visits (from baseline)^d^**  **Median (Interquartile Range)** | **Day 30** | 148 | 0.00 (0.00, 0.00) | 91 | 0.00 (0.00, 0.00) | 0.60 |
|  | **Day 60** | 163 | 0.00 (0.00, 0.00) | 101 | 0.00 (0.00, 0.00) | 0.32 |
|  | **Day 120** | 163 | 0.00 (0.00, 0.00) | 103 | 0.00 (0.00, 0.00) | 0.25 |
| **Number of Specialist Visits (from baseline)^d^**  **Median (Interquartile Range)** | **Day 30** | 148 | 0.00 (0.00, 0.00) | 91 | 0.00 (0.00, 0.00) | 0.44 |
|  | **Day 60** | 163 | 0.00 (0.00, 0.00) | 101 | 0.00 (0.00, 0.00) | 0.17 |
|  | **Day 120** | 163 | 0.00 (0.00, 0.00) | 103 | 0.00 (0.00, 0.00) | 0.26 |
| ^a^Intent-to-treat data are presented as incidence, Least Squared Mean (LSM) ± Standard Error of the Mean (SEM), or median number; ^b^Analyzed by chi-square test; ^c^Analyzed by ANOVA(study center, study group, gender); ^d^Analyzed by Wilcoxon rank sum test ; HCP: Healthcare Professional; DC: dietary counseling; ONS+DC: oral nutrition supplement + dietary counseling; bolded *P*-values indicate significance | | | | | | |

**Supplementary Table 10. Caregiver Perception^a^**

|  |  | **ONS+DC** | **DC** | **Effect Size** | ***P*-value** |
| --- | --- | --- | --- | --- | --- |
| **“Since using the [study product/nutrition changes recommended by the study dietitian], I feel more confident that my child is getting the right nutrition for his or her growth and development” (0-100)^b^** | **LSM ± SEM** | 83.05 ± 2.18 | 80.04 ± 2.49 | -3.016±2.638 | 0.25 |
|  | **n** | 126 | 80 |  |  |
| **“How satisfied were you with your child’s growth while using [the study product/the recommended nutrition changes]?”, n (%)^c^** | **Very Unsatisfied** | 1 (0.8) | 0 (0.0) |  | 0.08 |
|  | **Somewhat Unsatisfied** | 3 (2.3) | 3 (3.8) |  |  |
|  | **Neither Satisfied or Unsatisfied** | 10 (7.8) | 10 (12.5) |  |  |
|  | **Somewhat Satisfied** | 29 (22.7) | 28 (35.0) |  |  |
|  | **Very Satisfied** | 85 (66.4) | 39 (48.8) |  |  |
| **“How likely would you be to recommend the [study product/the nutrition changes] to your friends who may be concerned about their child’s growth?, n (%)^c^** | **Very Unlikely** | 0 (0.0) | 1 (1.3) |  | **0.003** |
|  | **Somewhat Unlikely** | 0 (0.0) | 1 (1.3) |  |  |
|  | **Neither Unlikely or Likely** | 3 (2.3) | 9 (11.3) |  |  |
|  | **Somewhat Likely** | 24 (18.8) | 21 (26.3) |  |  |
|  | **Very Likely** | 101 (78.9) | 48 (60.0) |  |  |
| ^a^Intent-to-treat data are presented as percent of respondents; ^b^Analyzed by ANOVA (study center, gender, study group, study group*gender, age group, study group*age group); ^c^Analyzed by Fisher’s exact test; DC: dietary counseling; ONS+DC: oral nutrition supplement + dietary counseling; bolded *P*-values indicate significance | | | | | |
